# How Users Perceive and Respond to Digital Humans: A Scoping Review of Agency, Embodiment, Social Role, and Response Outcomes

**DOI:** 10.3390/bs16091700

**Published:** 2026-09-20

**Authors:** Xuandi Gong, Li Zeng

**Affiliations:** 1 School of International Cultural and Communication, Communication University of Zhejiang, Hangzhou 310018, China; gxd0720@cuz.edu.cn; 2 Department of Methodology and Statistics, Tilburg School of Social and Behavioral Sciences, Tilburg University, 5037 DB Tilburg, The Netherlands

**Keywords:** digital humans, emerging media, user perception, user responses, scoping review

## Abstract

Digital humans are emerging as a form of mediated communication, appearing as virtual influencers, virtual agents, avatars, VTubers, AI companions, and other humanlike digital entities across social media, entertainment, commerce, and online communities. Research on how users perceive and respond to these entities remains fragmented across disciplines. This scoping review synthesizes evidence on users’ perceptions of and responses to digital humans. Following established scoping review guidance and PRISMA-ScR reporting principles, we searched Scopus, Web of Science, and EBSCO for English-language, peer-reviewed studies published between 2016 and 2025. Fifty-one studies met the inclusion criteria and were synthesized descriptively and thematically. The synthesis identified three recurring dimensions of user perception (i.e., perceived agency, perceived embodiment, and perceived social role) and three broad response domains: psychological, relational, and behavioral. These findings suggest that users’ responses to digital humans are shaped by more than human-likeness alone, with capability, embodied presence, and social role also emerging as important interpretive dimensions. The evidence base was concentrated in marketing and commerce, with limited attention to broader media contexts, long-term interaction, cultural diversity, and negative or ambivalent responses. Overall, this review provides an evidence-organizing framework for understanding how users interpret, relate to, and act toward digital humans.

## 1. Introduction

Digital humans are increasingly embedded in contemporary digital media environments, including social media, marketing communication, service interfaces, education, entertainment, and human computer interaction (Cui & Liu, 2023; Qu et al., 2025). Rather than constituting a single standardized form, digital humans encompass a heterogeneous range of humanlike digital entities that differ in their degree of embodiment, autonomy, interaction, and social or functional roles (Sorosrungruang et al., 2024; X. Wang et al., 2024). They appear in several recognizable forms. Virtual influencers, such as the computer-generated imagery character Lil Miquela, operate on social media platforms, accumulate followers, and collaborate with brands, making them particularly visible in influencer and commercial communication (Block & Lovegrove, 2021; Drenten & Brooks, 2020). Virtual agents, including chatbots and voice assistants, are used in customer service, education, and everyday interaction, where perceived social presence can shape rapport and engagement (Krämer et al., 2018; Purington et al., 2017). Avatars and VTubers also represent important forms, as animated or stylized personas perform and interact with audiences, blurring the boundary between performer and character (Lu et al., 2021; Zhou, 2020).

For behavioral research, the significance of digital humans lies not only in how they are technically produced, but also in how users perceive and respond to them (M. M. Barari et al., 2026). A digital human may be visibly artificial and still be experienced as socially present, trustworthy, entertaining, attractive, uncanny, persuasive, or emotionally meaningful (Cao et al., 2026; Sorosrungruang et al., 2024). Users may recognize that a virtual influencer is computer-generated, that a VTuber is represented through a virtual avatar, or that a chatbot is an artificial conversational agent, yet still evaluate and respond to these entities in social and relational terms, including perceived authenticity, credibility, trust, attachment, parasocial connection, and relational closeness (Koles et al., 2024; Lim & Lee, 2023; F. Liu & Wang, 2025; Skjuve et al., 2022). As mediated sources, interactive interfaces, content performers, and increasingly social partners, digital humans occupy communicative roles that extend beyond conventional technological artifacts (Audrezet et al., 2025; Yuan et al., 2026). The Computers Are Social Actors (CASA) paradigm provides a theoretical basis for understanding this phenomenon by proposing that individuals may apply social rules, expectations, and interpersonal responses to computers and interactive technologies even when they are aware that these entities are not human (Nass & Moon, 2000; Nass et al., 1994). Subsequent research has extended this logic to virtual and conversational agents, demonstrating that humanlike social cues can elicit social responses, while sustained interactions with artificial agents can also give rise to relational processes (Klein, 2025; Qu et al., 2025; Skjuve et al., 2022). Through this perspective, digital humans can function as social actors not because users necessarily mistake them for real people, but because humanlike and social cues can prompt users to apply interpersonal rules, expectations, and modes of interaction to artificial agents (M. Kim & Baek, 2024; Nass et al., 1994). CASA therefore helps explain why users may recognize the artificial nature of digital humans while nevertheless forming social evaluations, expectations, and relational responses toward them.

Existing research has generated important insights, but its findings remain difficult to integrate because digital humans are studied under different labels, in different contexts, and through different conceptual traditions. In computer science and engineering, they are often treated as technological artifacts, with attention to realism, functionality, technical capability, and uncanny effects (R. F. Khan & Sutcliffe, 2014; Magistris et al., 2015; Seymour et al., 2021). In marketing and advertising, they are commonly examined as virtual influencers or persuasive sources, with a focus on credibility, authenticity, engagement, brand evaluation, and purchase intention (Lou, 2022; Thomas & Fowler, 2021). In human–computer interaction, they are frequently studied as interactive or embodied agents through concepts such as anthropomorphism, social presence, responsiveness, and the Computers Are Social Actors paradigm (Nass & Moon, 2000; Van Pinxteren et al., 2023). Related entities may therefore be framed as technological agents, mediated sources, performers, or social partners, while adjacent terms such as digital human, virtual human, virtual influencer, avatar, VTuber, AI companion, embodied conversational agent, and chatbot are sometimes used separately and sometimes overlap.

This fragmentation creates two related problems. First, conceptually similar user perceptions and responses are often described using different constructs across disciplines and entity types. Second, the available evidence has not yet been systematically mapped in a way that clarifies how these constructs relate to broader patterns of user perception and response. As a result, it remains difficult to compare findings across contexts or to identify where the evidence base is concentrated and where important gaps remain.

A scoping review is particularly appropriate for this purpose because the aim is to map the breadth and characteristics of a heterogeneous and conceptually fragmented literature, clarify how key concepts have been studied, and identify gaps in the existing evidence rather than estimate a pooled effect or evaluate the effectiveness of a specific intervention (Levac et al., 2010; Peters et al., 2021; Tricco et al., 2018). Accordingly, this scoping review examines how users’ perceptions of and responses to digital humans have been studied across the existing literature. Entity types, research contexts, and methodological approaches are used to characterize the evidence base, while the synthesis identifies recurring perception concepts, response outcomes, and research gaps (Levac et al., 2010; Tricco et al., 2018). Perception and response served as broad organizing concerns from the outset, while the specific dimensions presented in this review were developed through iterative comparison and synthesis of the constructs reported in the included studies. Here, these dimensions are used as broader organizing categories that bring together conceptually related study-specific constructs; they are not intended as prespecified theoretical variables or as a fixed taxonomy.

Accordingly, the review addresses the following research questions:

RQ1: How has existing research conceptualized users’ perceptions of digital humans?

RQ2: What types of user responses to digital humans have been examined?

RQ3: What conceptual, methodological, and empirical gaps remain in research on how users perceive and respond to digital humans?

The remainder of the article is organized as follows. Section 2 describes the review methods, including eligibility criteria, search strategy, study selection, data charting, and data synthesis. Section 3 presents the characteristics of the included studies, maps user perception dimensions and response outcomes, integrates the relationships between them, and identifies gaps in the current evidence base. Section 4 discusses the main findings and their theoretical implications, limitations, and directions for future research, followed by the conclusion in Section 5.

## 2. Methods

### 2.1. Eligibility Criteria

Studies were included in the scoping review if they met the following criteria:

(a) Written in English and published in peer-reviewed academic journals or conference proceedings;
(b) Focused on users’ perceptions of or responses to digital humans (including but not limited to studies employing alternative terms or designations for digital humans);
(c) Published between 2016 and 2025.

The following types of publications were excluded: non-English publications, reviews, book chapters, author responses, and correction notices.

### 2.2. Search Strategy

Given the interdisciplinary nature of digital human research, Scopus, Web of Science, and EBSCO were selected to provide complementary coverage across relevant disciplines (Gusenbauer & Haddaway, 2020). Combining multiple databases helps reduce the risk of missing relevant studies because their indexing and retrieval coverage can differ substantially (Wanyama et al., 2022).

The literature search used the “title” and “abstract” fields (or their database-specific equivalents). The search strategy combined terms referring to digital humans and related entities with terms concerning user perceptions and responses. Searches were conducted separately for perception-related and response-related terms across the three databases. Where supported by the database, filters corresponding to the eligibility criteria described in Section 2.1 were applied at the search stage, including language, publication period, and publication type. The complete source-specific search strategies are provided in Supplementary Table S3.

The search identified 6216 records in Scopus. After restricting the document type to articles and conference papers, the source type to journals and conference proceedings, the language to English, and the publication stage to final publications, and retaining subject areas relevant to the research question, 3878 records were retained. The Web of Science search initially identified 4954 records; after applying screening criteria related to document type, language, and subject category, 3807 records remained. In EBSCO, we identified 353 records. In total, 8038 records were identified across the three databases.

### 2.3. Study Selection

Study selection followed established scoping review guidelines (Arksey & O’malley, 2005). This procedure consisted of two sequential stages: (i) title and abstract screening, followed by (ii) full-text eligibility assessment of potentially relevant studies. At each stage, two authors independently screened the identified records, and disagreements regarding eligibility were resolved through discussion and consensus (Peters et al., 2021). Inter-rater agreement was calculated at the title-and-abstract screening stage (88.5%) and full-text screening stage (92.2%).

Among the 8038 records identified across the three databases, 5770 duplicates were removed, leaving 2268 records for screening. A further 704 records were excluded manually according to the predefined bibliographic eligibility criteria, including publication type (e.g., non-journal articles or non-conference papers), language (non-English), study design (e.g., systematic reviews or literature reviews), and other predefined exclusion conditions, leaving 1564 records.

To improve screening efficiency, a keyword-based exclusion step was applied to remove studies primarily related to robotics or healthcare contexts (e.g., robot, medical care, health, health programs, patient, healthcare, treatment, cure, and therapy), reducing the dataset to 1480 records. The remaining records were subsequently assessed using two review-specific eligibility criteria developed for this concept-oriented scoping review: (i) whether the study focused on digital humans or closely related technologies (e.g., virtual humans, AI avatars, or virtual influencers), and (ii) whether it investigated users’ perceptions, evaluations, experiences, or responses toward these technologies. Application of these two criteria reduced the number of records to 942 and subsequently 497 studies, respectively.

The remaining 497 records then underwent a more detailed manual assessment of titles and abstracts to evaluate their conceptual relevance to the review objectives. At this stage, studies that primarily addressed technical development, algorithm design, rendering techniques, system architecture, or other engineering-oriented topics without providing evidence on user perceptions or user responses were excluded. This process resulted in 125 studies eligible for full-text retrieval. Full texts could not be obtained for 27 studies, leaving 98 articles for full-text eligibility assessment.

**Figure 1:**
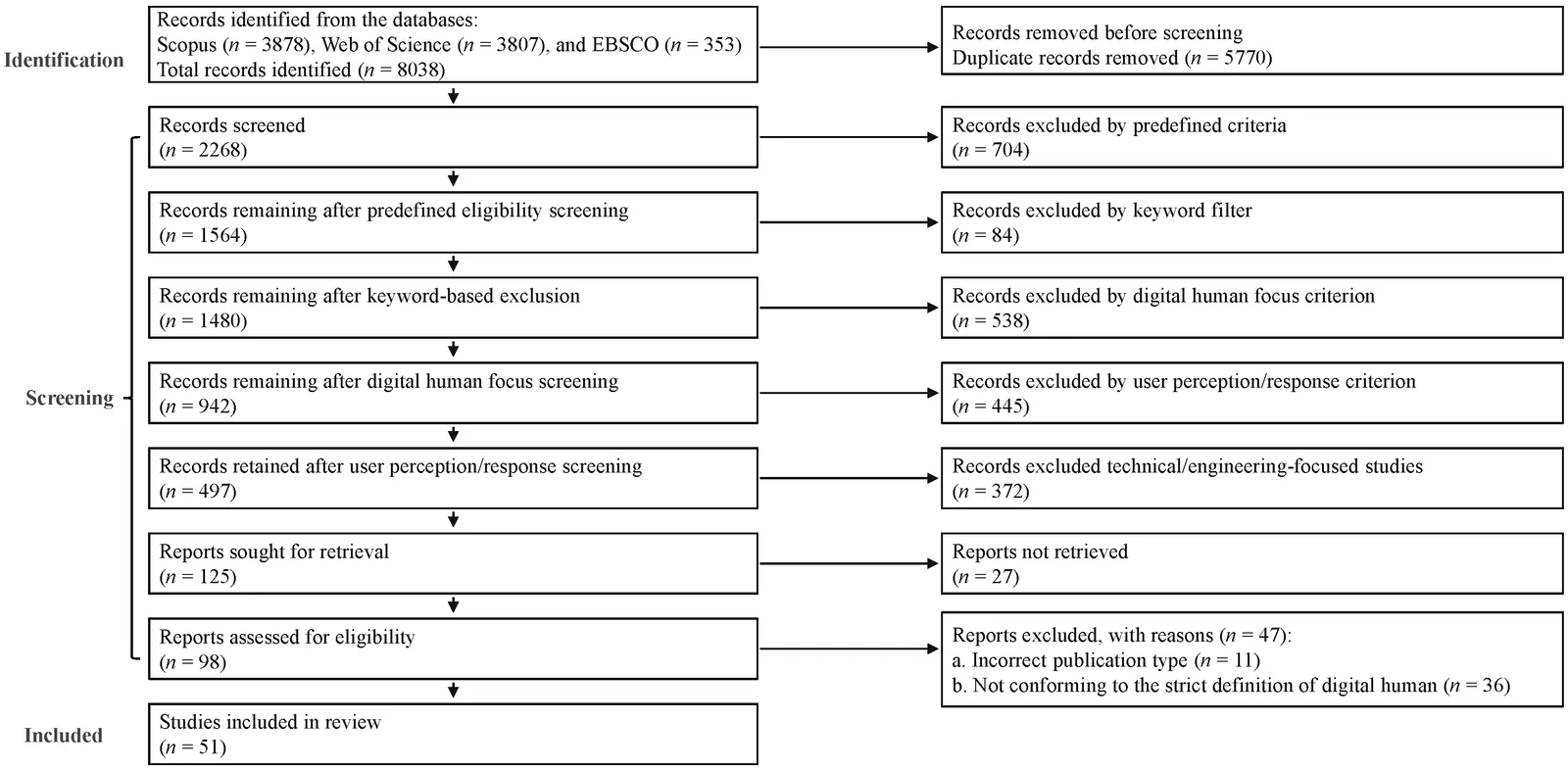
PRISMA flow diagram of the study selection process.

During the full-text review, the remaining studies were evaluated against the predefined inclusion and exclusion criteria to confirm their eligibility. This assessment verified the study focus, the presence of sufficient evidence on user perceptions or responses, and the availability of extractable information relevant to the review objectives. Ultimately, 51 studies met all eligibility criteria and were included in the final synthesis. The complete study selection process is presented in Figure 1.

### 2.4. Data Charting

A data charting form was developed to extract and organize information from the included studies. The form contained eight fields: Author (Year), Entity Label, Context, Study Design, User Perception Dimensions, User Response Outcomes, Key Findings, and Limitations. The data charting form and the accompanying coding guidance are provided in Supplementary Table S2, and the completed study-level charting and coding results are presented in Supplementary Table S1.

For data charting, both authors independently extracted and coded information from the included studies. Wherever possible, the entity labels, perception concepts, and response outcomes used in the original studies were retained. Any disagreements or uncertainties arising during coding were resolved through discussion and consensus between the two authors.

### 2.5. Data Synthesis

Given the diversity of concepts, measures, and study designs across the included studies, we synthesized the data descriptively and thematically to identify recurring patterns across the literature. First, we summarized the characteristics of the included studies, including publication year, country or region, research context, method, and entity labels. These descriptive results were used to characterize how research on users’ perceptions of and responses to digital humans has developed across fields and contexts.

Second, we mapped the terminology used to describe digital humans and adjacent humanlike digital entities. Because the literature uses overlapping labels such as digital human, virtual human, virtual influencer, avatar, VTuber, AI companion, embodied conversational agent, virtual agent, and chatbot, the synthesis retained the labels used in the original studies while identifying points of overlap and divergence.

Third, the coded perception concepts and response outcomes were compared across studies and synthesized into broader organizing categories. Conceptually related constructs were grouped based on their meanings, usage, measurement, and theoretical framing in the original articles. Perception concepts were grouped according to the types of interpretive judgments users made about digital humans, whereas response outcomes were grouped according to the types of psychological, relational, or behavioral reactions examined in the literature.

The grouping process was iterative and interpretive. Categorical assignments were refined through comparison across studies and discussion between the two authors, with attention to how concepts were used, measured, or theoretically positioned in the original articles. This process was used to identify recurring patterns, similarities and differences in user perceptions and responses, and areas that remained comparatively underexplored. The resulting synthesis informed the mapping tables linking entity labels, user perception concepts, user response outcomes, and gaps in the literature.

No formal critical appraisal of the included studies was conducted because the purpose of this scoping review was to map and synthesize the available evidence rather than to evaluate study quality. This review was retrospectively registered on the Open Science Framework (OSF; https://doi.org/10.17605/OSF.IO/M2GQF).

## 3. Results

### 3.1. Characteristics of Included Studies

**Figure 2:**
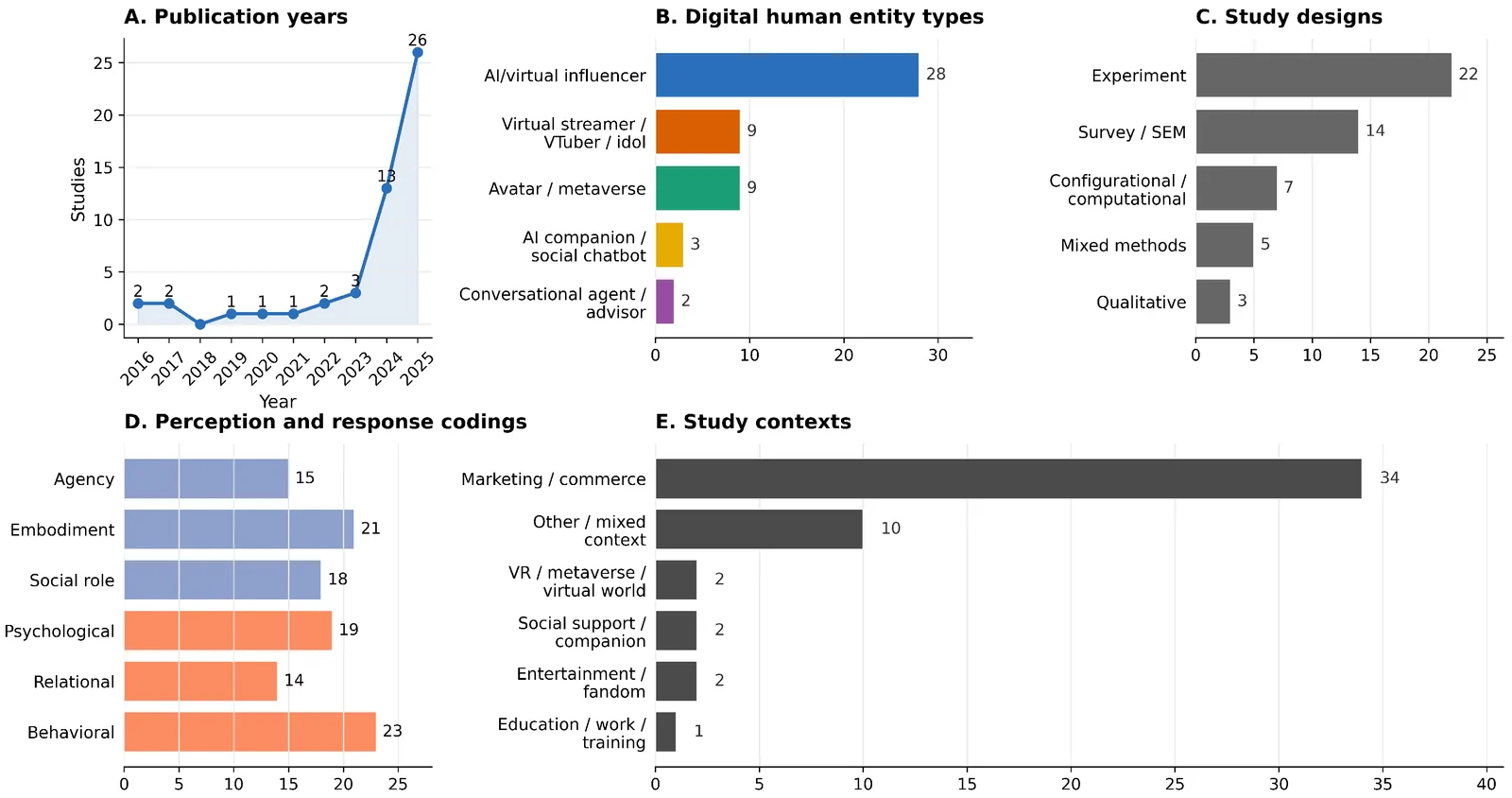
Characteristics of the 51 included studies. (**A**) Number of publications by year; (**B**) distribution by digital human entity type; (**C**) distribution by study design; (**D**) frequency of perception-dimension and response-domain codings; and (**E**) distribution by study context.

The final corpus included 51 studies published between 2016 and 2025. As shown in Figure 2A, research on user perceptions and responses to digital humans remained relatively limited before 2023, with only sporadic publications between 2016 and 2022. A sharp increase was observed in the most recent period, with 13 studies published in 2024 and 26 studies published in 2025.

The included studies were unevenly distributed across entity types, study designs, and application contexts. AI and virtual influencers were the most frequently examined entities (n=28), followed by virtual streamers, VTubers, or virtual idols (n=9) and avatar- or metaverse-related entities (n=9). AI companions or social chatbots (n=3) and conversational agents or advisors (n=2) were less frequently represented (Figure 2B).

Experimental designs were most common (n=22), followed by survey- or SEM-based studies (n=14). Smaller numbers of studies used configurational or computational approaches (n=7), mixed methods (n=5), or qualitative designs (n=3) (Figure 2C).

Figure 2D summarizes the perception and response categories assigned during synthesis. Because individual studies could address more than one perception dimension or response domain, these coding counts are not mutually exclusive and may exceed the total number of included studies. Perceived embodiment was the most frequently identified perception dimension (n=21), followed by perceived social role (n=18) and perceived agency (n=15). Behavioral responses were the most frequently examined response domain (n=23), followed by psychological (n=19) and relational responses (n=14).

The evidence base was also concentrated in particular application contexts. Marketing and commerce accounted for 34 studies, whereas social support or companionship (n=2), VR, metaverse, or virtual-world contexts (n=2), entertainment or fandom (n=2), and education, work, or training (n=1) were much less common. Ten studies were classified as involving other or mixed contexts (Figure 2E). Overall, the descriptive distribution indicates that the current evidence base is shaped predominantly by consumer-facing and platform-mediated settings.

**Figure 3:**
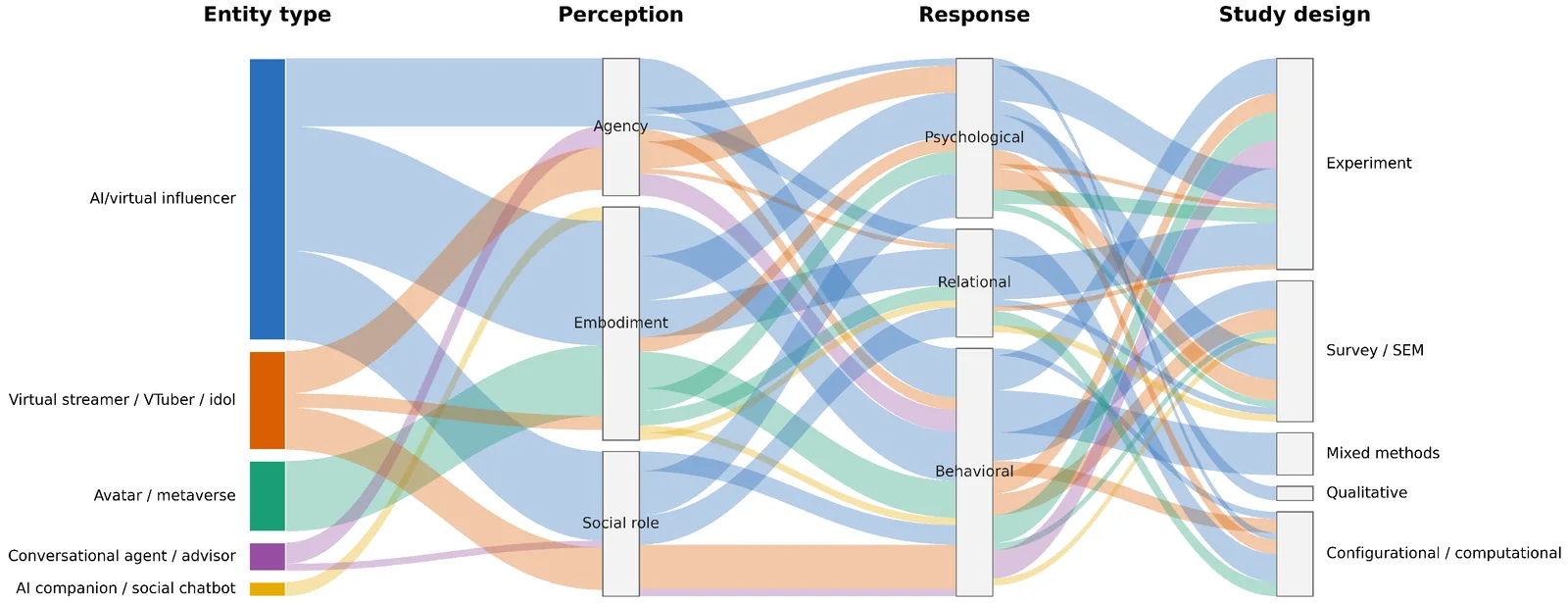
Evidence flow across digital human entity types, perception dimensions, response domains, and study designs. The width of each flow represents the relative number of coded connections across the included studies.

Figure 3 shows how these distributions are reflected in the structure of the evidence. AI and virtual influencer studies contributed the largest share of connections across all three perception dimensions, with particularly visible links through perceived embodiment and perceived social role to psychological and behavioral responses. Virtual streamer, VTuber, and virtual idol studies were more visibly connected to perceived agency and social role, whereas avatar- and metaverse-related studies were concentrated more strongly around embodiment. Conversational agents, advisors, AI companions, and social chatbots formed a smaller strand, with visible connections through agency and social role to behavioral responses. Taken together, Figure 2 and Figure 3 show that the recent growth of digital human research has occurred largely in consumer-facing applications, while smaller bodies of work have begun to examine digital humans as interactive partners, companions, advisors, and socially represented others.

### 3.2. Mapping User Perception Dimensions

User perception is a central theme in research on digital humans because it concerns how users interpret and evaluate these entities in interaction. Across the reviewed studies, users made sense of digital humans through their perceived capabilities, embodied presence, and relational position. We therefore organized user perception into three interrelated dimensions: perceived agency, perceived embodiment, and perceived social role. These dimensions capture how users interpret digital humans as capable of action, perceptually and socially present, and positioned within social interaction. Rather than imposing a fixed taxonomy, this framework synthesizes recurring perception concepts across studies. Table 1 summarizes the three organizing dimensions, their core questions, common concepts, and representative studies. Methodological approaches across the included studies are reported at the study level in Supplementary Table S1.

#### 3.2.1. Perceived Agency

Perceived agency refers to the extent to which users interpret digital humans as capable of acting, responding, adapting, or expressing intention. Across the reviewed studies, agency was examined through constructs such as perceived intelligence, competence, autonomy, interactivity, responsiveness, communicative capability, animism, and mind perception (Esfahani et al., 2020; Jung et al., 2022; T. Kim et al., 2025; Ranjbartabar et al., 2021; Zhang & He, 2025; Zourrig et al., 2025). Although these constructs differ in theoretical origin, they converge on a common interpretive question: whether the digital human is experienced as an active entity whose behavior can be treated as meaningful action rather than as a passive digital representation.

Perceived intelligence and competence were particularly prominent in recommendation- and task-oriented settings. Studies of AI influencers and virtual streamers examined whether these entities appeared knowledgeable, capable, and able to provide useful guidance. Perceived competence was associated with consumers’ evaluations of products endorsed by AI influencers, while perceived intelligence was examined as a characteristic shaping responses to virtual streamers (Na et al., 2025; Toyib & Paramita, 2025). These studies positioned competence as a practical judgment about whether a digital human could credibly perform a task or communicative function.

Autonomy and interactivity concerned how independently and responsively digital humans appeared to operate. Studies distinguished more autonomous AI influencers from entities understood as externally managed and examined autonomy and interactivity as affordances associated with perceived humanness, trust, and engagement (C. T. Lee & Shen, 2025; Sands et al., 2022). Interactional capacity was also operationalized differently across contexts. In virtual-streaming commerce, product-oriented and social-oriented interaction were linked to different evaluative and behavioral pathways, while responsiveness was associated with purchase intention through social presence (X. Liu & Zhang, 2024). These findings do not establish a single agency pathway, but they show how users’ judgments of capability are informed by whether a digital human can respond in ways that appear relevant to the task and interaction context.

Research on AI companions extended this issue toward trainability and user-mediated adaptation. Pan et al. (2025) documented how Replika users invested intellectual and emotional labor in training and personalizing the chatbot. In this review, such practices are treated as relevant to agency because they show how a digital human’s apparent behavioral capacity may be shaped jointly by system functions and continuing user input, rather than being experienced as entirely fixed.

Some studies examined agency through deeper attributions of aliveness or mind. Perceived animism described the extent to which humanlike digital entities appeared animate or alive, whereas agentic mind perception concerned whether users attributed intention and goal-directed mental capacity to them (Toyib & Paramita, 2024; Zhang & He, 2025; Zourrig et al., 2025). Lower attribution of agentic mind to AI influencers was associated with lower brand trustworthiness and purchase intention (Zhang & He, 2025). These findings extend agency beyond observable performance to the question of whether users perceive an underlying capacity for intentional action.

Across these studies, agency operated at two related levels. One concerned observable or inferred functional capability—for example, competence, responsiveness, and interactivity—whereas the other concerned stronger attributions of autonomy, intentionality, or mind. This distinction helps explain why agency-related judgments appeared in both task-oriented evaluations and more social interpretations of digital humans. Perceived agency is therefore better understood here as a user judgment about meaningful action capacity than as a technical property of the system itself.

#### 3.2.2. Perceived Embodiment

Perceived embodiment refers to how a digital human becomes perceptually and socially present to users through appearance, expression, communication, and other sensory or representational cues. The included studies approached this process through anthropomorphism, human-likeness, realism, social presence, expressive behavior, authenticity, and creepiness. These constructs do not describe a single continuum of visual realism; rather, they capture different ways in which a digital representation is experienced as an embodied and socially meaningful presence.

Anthropomorphism and human-likeness were among the most frequently examined embodiment-related concepts (Dabiran et al., 2024; M. B. Khan et al., 2025; X. Liu & Zhang, 2025; Toyib & Paramita, 2024), but greater human resemblance did not produce uniformly stronger or more favorable responses. Avatar human-likeness was associated with psychological closeness and favorable responses in virtual-reality settings (Chae et al., 2025), and different virtual influencer forms generated different levels of social presence and emotional attachment (Yan et al., 2024). At the same time, virtual influencers could elicit parasocial interaction despite lower perceived humanness and similarity (Stein et al., 2024). Greater anthropomorphism was also associated with increased engagement but reduced consumer well-being through upward social comparison (M. Barari et al., 2025), while combinations of realism and animacy could evoke eeriness and reduce trustworthiness in avatar-mediated interaction (Shin et al., 2019). These findings indicate that embodiment effects depend on how humanlike cues are interpreted rather than on resemblance alone.

A second strand of research concerned experienced social presence. Talking avatars and enhanced avatar expressions increased the sense that another social entity was present in the mediated environment (Liew et al., 2017; Oh et al., 2016). In virtual-streaming and influencer contexts, anthropomorphic appearance or responsiveness was likewise connected with social presence, which could then be associated with attachment, perceived value, or behavioral intention (X. Liu & Zhang, 2024, 2025; Yan et al., 2024; Zourrig et al., 2025). Social presence occupied different analytical positions across primary studies; it is treated as part of perceived embodiment here when it describes how socially present the digital human itself was experienced to be.

Embodiment also involved judgments of coherence and authenticity rather than appearance alone. Koles et al. (2024) identified true-to-ideal, true-to-fact, and true-to-self manifestations of virtual influencer authenticity, while M. Kim and Baek (2024) showed that similarity cues could shape both authenticity and creepiness judgments. Thompson et al. (2025) further found that moral and cognitive anthropomorphism contributed to perceived shared reality and engagement, illustrating that the meanings attributed to a digital human can extend beyond physical resemblance. Taken together, these findings suggest that users evaluate whether appearance, expression, identity, and communicative behavior form a coherent mediated persona within a particular context.

Overall, perceived embodiment is not reducible to maximizing human-likeness. The reviewed studies instead point to a combination of representational form, expressive and communicative cues, experienced presence, and contextual coherence. This helps account for the mixed pattern in which embodied humanlike cues could support closeness, social presence, and positive evaluation in some settings while contributing to eeriness, comparison, distrust, or reduced authenticity in others.

#### 3.2.3. Perceived Social Role

Perceived social role refers to the functional and relational position users attribute to a digital human within a particular interaction context. This dimension is analytically different from entity type: an entity label identifies what kind of digital form is being studied, whereas social role concerns what users understand that entity to be doing in the interaction and what expectations follow from that position. Across the reviewed literature, digital humans were positioned as influencers, streamers, idols, shopping presenters, advisors, support providers, companions, and avatar-mediated partners.

Influencer identity was the most prominent social role in the included literature. AI and virtual influencers were examined as brand endorsers, campaign figures, and sources of product-related communication (Toyib & Paramita, 2024; van Berlo & Breves, 2025). Their reception depended on factors including perceived credibility, interactivity, influencer–product congruence, and fit with the broader campaign or brand context (Hidayat et al., 2024; Y. Wang et al., 2025). In luxury branding, the effects of virtual and human influencers also varied according to their alignment with brand-heritage narratives (Mo & Wang, 2025). These studies indicate that users evaluated the influencer role through both its persuasive function and its contextual legitimacy.

Streamer, VTuber, and virtual idol roles were shaped by performance practices and platform participation. Research on VTuber concerts showed that audiences could participate both as spectators and as creators in constructing the meaning of the performer and fandom experience (S. Lee & Lee, 2023; Li, 2023). In live-streaming commerce, virtual streamers were positioned as shopping presenters whose acceptance was associated with anthropomorphism, psychological distance, trust, and product type (Chen et al., 2024). Users also assessed virtual streamers in relation to human counterparts, with switching intention shaped by innovation resistance, shopping motivations, and personality characteristics (Shao, 2025). Streamer-role perception therefore involved both recognition of a distinctive mediated performer and evaluation of whether the entity could fulfil functions conventionally performed by humans.

Digital humans were also positioned as providers of support or companionship. T. Kim et al. (2025) compared virtual and human influencers in cognitive and emotional support contexts and examined differences in support efficacy, perceived interactivity, and social attractiveness. In AI companion research, users interpreted Replika through language, visual presentation, and broader ideas about sociality and personhood, while also participating in training and personalizing the system (Pan et al., 2025; Rocha, 2025). These studies show that companion and support roles were negotiated through continuing interaction rather than determined solely by system functionality (Ranjbartabar et al., 2021).

Related evidence appeared in avatar-mediated interpersonal interaction. Chesney et al. (2016) examined how user personality and social value orientation influenced avatar-mediated friendship. Although avatars in this setting were not autonomous AI companions, the study illustrates how the perceived role of a digitally represented partner can depend on the characteristics and expectations of the human participants.

Homophily and similarity cut across these role categories. Studies examined whether shared attitudes, interests, identities, or communicative styles made virtual influencers appear more relatable or credible (Alboqami, 2023; Yoo et al., 2025). Language similarity, interest similarity, and attitudinal homophily were also associated with authenticity and creepiness judgments (M. Kim & Baek, 2024). Here, similarity is treated as role-related when it influences whether a digital human is recognized as a relevant source, performer, or relational partner. Moral and cognitive anthropomorphism could further contribute to shared reality and the meanings users constructed around virtual influencer identities (Thompson et al., 2025).

Across these studies, role perception provided a contextual frame for interpreting other cues. Competence was consequential when a digital human was expected to advise or recommend; similarity and credibility mattered when it functioned as an influencer or relational partner; and appearance or interactivity acquired different meanings depending on whether the entity was encountered as a performer, shopping presenter, advisor, or companion. Perceived social role therefore captures the expectations against which agency and embodiment cues become appropriate, credible, or meaningful in a given mediated interaction.

### 3.3. Mapping User Response Outcomes

User response refers to the evaluative, affective, relational, and action-oriented outcomes examined in relation to digital humans. Whereas the perception dimensions organize what users attribute to or infer about a digital human, the response domains organize how users evaluate the encounter, experience social connection, or act and intend to act. Across the reviewed literature, responses were grouped into three interrelated domains: psychological, relational, and behavioral. These categories are analytical rather than temporal; the synthesis does not assume that one domain necessarily precedes another. Table 2 summarizes the three response categories, common outcomes, and representative studies.

#### 3.3.1. Psychological Responses

Psychological responses refer to users’ evaluative and affective reactions toward digital humans, including trust, credibility, authenticity judgments, empathy, enjoyment, discomfort, and other positive or negative appraisals. In many studies, these responses were examined as proximal outcomes of digital human characteristics or as mechanisms associated with later relational or behavioral outcomes, although the synthesis does not assume a fixed sequence.

Trust and credibility were among the most frequently examined psychological responses. Research on AI and virtual influencers showed that trust could arise from configurations of source attractiveness, credibility, congruence, and other cues rather than from a single attribute (Alboqami, 2023; Melnychuk et al., 2024). Trust-related evaluations were also sensitive to disclosure: revealing or emphasizing a virtual influencer’s non-human nature could alter anthropomorphism, credibility, and brand trust (Muniz et al., 2024; van Berlo & Breves, 2025). At a deeper attributional level, lower perceived agentic mind in AI influencers was associated with lower brand trustworthiness and purchase intention (Zhang & He, 2025). In live-streaming commerce, responsiveness and interaction were connected with purchase-related outcomes through social presence and perceived value (H. Liu et al., 2025b; X. Liu & Zhang, 2024). Across these studies, psychological evaluations often served as mechanisms through which agency, embodiment, or role-related cues became consequential for later outcomes.

Affective responses included empathy, enjoyment, flow, affection, and emotional engagement. Smiling expressions influenced affective and cognitive empathy as well as brand-authenticity, purchase, and following outcomes, with effects varying by advertising context (Jiang et al., 2025). Virtual-streamer characteristics were also linked to trust and affection (Na et al., 2025), while studies of virtual influencer endorsement examined emotional engagement and flow in relation to aesthetic, entertainment, credibility, and parasocial factors (H. Liu et al., 2025a; Vo et al., 2025; Zhang et al., 2025). These findings show that users’ responses involved not only judgments about whether a digital human was credible or useful, but also affective experiences generated by content, expression, and interaction.

Authenticity was coded as a psychological response when the primary study treated it as a user evaluation rather than as an attributed property of the digital human. Qualitative work identified multiple manifestations of virtual influencer authenticity (Koles et al., 2024), while similarity cues were associated with authenticity and creepiness judgments (M. Kim & Baek, 2024). Negative psychological responses included eeriness, distrust, and reduced well-being. Uncanny or incongruent avatar representations could reduce trustworthiness and shape friendship decisions (Shin et al., 2019), whereas greater virtual influencer anthropomorphism was associated with higher engagement but lower consumer well-being through upward social comparison (M. Barari et al., 2025). The psychological domain therefore includes both favorable and unfavorable interpretations of the same broad class of humanlike and social cues.

Overall, psychological responses often occupied an intermediate analytical position between characteristics attributed to a digital human and later relational or behavioral outcomes, but this role was not uniform across studies. Trust, authenticity, empathy, enjoyment, and discomfort were sometimes endpoints and sometimes explanatory mechanisms, reinforcing the need to distinguish the substantive meaning of a construct from the position it occupies in a particular empirical model.

#### 3.3.2. Relational Responses

Relational responses refer to users’ perceived social connection and interpersonal involvement with digital humans. This category includes parasocial interaction, social presence, psychological closeness, perceived similarity, homophily, emotional attachment, and loyalty. These outcomes concern whether mediated encounters are experienced as socially meaningful relationships rather than only as evaluations of a technological or persuasive source.

Parasocial interaction was a central relational outcome in studies of virtual influencers. Perceived similarity and human-likeness shaped parasocial interaction with real and virtual influencers (Stein et al., 2024), while source expertise and trustworthiness were examined as antecedents of parasocial connection, engagement, and follower stickiness (Melnychuk et al., 2024). Other studies linked parasocial interaction to flow, engagement, loyalty, and purchase-related outcomes (Vo et al., 2025; Zhang et al., 2025). Across this work, parasocial constructs captured a form of social connection that could develop around mediated figures even when users were aware of their artificial or virtual status.

Social presence represented a more immediate sense that a digital human was socially available or “there” in the mediated interaction. Talking avatars and enhanced avatar expression increased social presence (Liew et al., 2017; Oh et al., 2016), while humanoid appearance and perceived animism were linked to social presence in virtual influencer research (Zourrig et al., 2025). Different virtual influencer forms also produced different levels of social presence and emotional attachment (Yan et al., 2024). Social presence was included in the relational domain when it represented a felt social connection; when primary studies treated it as an attributed characteristic or mediator, its classification followed that analytical role.

Psychological closeness and emotional attachment represented deeper forms of relational involvement. Avatar human-likeness was associated with psychological closeness in virtual-reality work and learning environments (Chae et al., 2025). Emotional engagement and attachment were also examined in virtual influencer endorsement and brand-relationship contexts (Dabiran et al., 2024; H. Liu et al., 2025a). In studies of Chinese Gen Z consumers, emotional attachment moderated some relationships between virtual influencer characteristics and purchase intention (Y. Wang et al., 2025). These studies positioned attachment both as a relational outcome and, in some models, as a condition shaping later behavioral responses.

Similarity and homophily cut across these relational processes. Perceived similarity was examined as an antecedent of parasocial interaction (Stein et al., 2024), while homophily and textual social cues were associated with trust and credibility in AI- and virtual influencer contexts (Alboqami, 2023; Yoo et al., 2025). Together, these studies suggest that perceived commonality can help digital humans become socially relevant, although similarity does not operate identically across contexts or outcomes.

Overall, the relational evidence spans different forms and depths of social involvement, from an immediate sense of social presence to parasocial connection, psychological closeness, attachment, and loyalty. These constructs are not interchangeable, but together they show that digital human encounters can become socially meaningful in ways that go beyond instrumental evaluation. At the same time, most evidence captures relatively short-term or cross-sectional forms of connection, leaving the development and dissolution of longer-term relationships comparatively underexamined.

#### 3.3.3. Behavioral Responses

Behavioral responses refer to users’ observable actions or behavioral intentions toward digital humans, their content, or associated products and services. Common outcomes included purchase intention, following intention, engagement, word-of-mouth, recommendation, adoption, switching intention, and avatar-based identity use. These outcomes were often examined alongside psychological and relational variables, but they were coded separately when they represented an intended or enacted action.

Purchase intention was the most frequently examined behavioral outcome, reflecting the strong marketing and commerce orientation of the evidence base. Studies linked purchase intention to virtual-streamer characteristics, credibility, flow, social presence, perceived value, and other evaluative or relational mechanisms (Dabiran et al., 2024; H. Liu et al., 2025b; X. Liu & Zhang, 2024; Xu et al., 2025; Zhang et al., 2025). Emotional expression also shaped purchase and following intentions under different advertising conditions (Jiang et al., 2025), while lower perceived agentic mind and brand trustworthiness constrained the effectiveness of AI influencer endorsements (S. S. Lee et al., 2025; Zhang & He, 2025). In metaverse-fashion research, avatar customization was associated with psychological ownership and purchase intention toward virtual products (D. Y. Kim & Jang, 2025; Xu et al., 2025).

Following intention and platform engagement reflected users’ willingness to maintain attention to or interact with digital humans. Emotional, relational, and entertainment-oriented content was associated with following and purchase intentions (H. Liu et al., 2025a; Rungruangjit et al., 2024). AI influencer affordances such as autonomy and interactivity were examined in relation to active engagement (C. T. Lee & Shen, 2025), while cognitive support and social attractiveness were also associated with engagement in virtual influencer contexts (T. Kim et al., 2025). Other studies showed that engagement could remain relatively strong despite lower trust in AI influencers (Sands et al., 2022), that moral and cognitive anthropomorphism could support shared reality and repeated engagement (Thompson et al., 2025), and that persona, disclosure, and posting characteristics predicted engagement with virtual influencer endorsements (Xia et al., 2025). Greater anthropomorphism could also increase engagement while simultaneously reducing well-being through upward social comparison (M. Barari et al., 2025), again showing that behavioral engagement is not equivalent to uniformly positive user experience.

Word-of-mouth and recommendation represented forms of behavioral diffusion. AI influencers could generate word-of-mouth intentions even when they were evaluated as less trustworthy than human influencers (Sands et al., 2022), while perceived homophily could support recommendation adoption through anthropomorphism (Toyib & Paramita, 2024). In VTuber research, parasocial interaction, attractiveness, personality, avatar design, and interaction patterns were associated with viewers’ decisions to watch and engage with livestreams (Li, 2023). Adaptive relational cues likewise encouraged continued responses to virtual advisors by improving the fit between interaction styles and user preferences (Ranjbartabar et al., 2021). These findings indicate that continued or diffusion-related behavior may arise through multiple evaluative and relational pathways rather than through trust alone.

Adoption and switching outcomes extended the evidence beyond immediate content engagement. In live-streaming commerce, anthropomorphism, psychological distance, trust, and product type were associated with willingness to accept virtual streamers (Chen et al., 2024), while AI influencers were also found to promote consumers’ adoption intentions toward AI-endorsed products by enhancing perceived product quality and trust (Toyib & Paramita, 2025). Switching from human to virtual streamers was examined in relation to innovation resistance, shopping motivations, and personality (Shao, 2025). Avatar research further examined the adoption of digital identities through perceived similarity, social presence, anthropomorphism, trust, enjoyment, customization, and psychological ownership (M. B. Khan et al., 2025; D. Y. Kim & Jang, 2025). Related studies also showed that users’ trust and intrinsic motivation influenced engagement and technology acceptance when interacting with conversational agents represented through different metaphors (Jung et al., 2022).

Overall, behavioral responses captured several distinct forms of action, but the evidence was dominated by purchase intention and platform engagement. Recommendation, switching, adoption, identity use, participation, and co-creation were represented less extensively, and many studies measured intentions rather than observed behavior over time. The behavioral literature therefore provides a comparatively strong account of immediate consumer-facing outcomes but a thinner account of sustained, non-commercial, or relationship-based forms of action.

### 3.4. Connecting Perceptions and Responses

**Figure 4:**
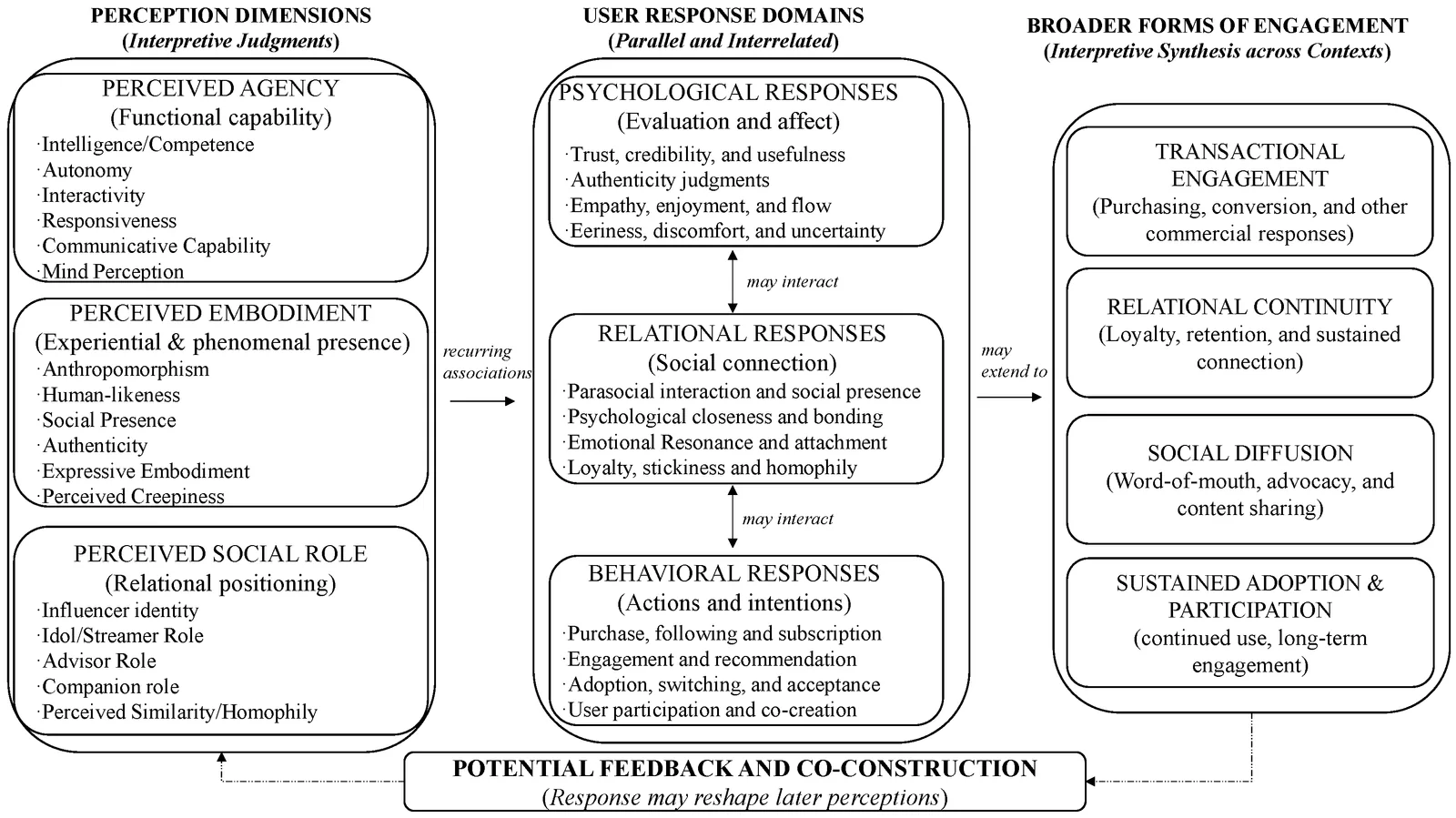
Integrative map of perception-response linkages in digital human research.

Figure 4 integrates the perception dimensions mapped in Section 3.2 with the response domains mapped in Section 3.3. The three perception dimensions represent related but non-equivalent interpretive problems. Agency concerns whether the digital human appears able to act meaningfully; embodiment concerns how it becomes perceptually and socially present; and social role concerns what kind of participant users understand it to be within the interaction. The response domains capture how those interpretations are accompanied by evaluation and affect, social connection, and intended or enacted behavior. The arrows summarize recurring associations across the included studies rather than universal causal or temporal pathways.

The theoretical approaches used in the primary studies help clarify why these dimensions are analytically distinct. Agency-oriented work drew on mind perception and competence-related perspectives to examine whether digital humans were attributed intentionality or task-relevant capability (Toyib & Paramita, 2025; Zhang & He, 2025), and affordance-oriented research examined autonomy and interactivity as perceived possibilities for action (C. T. Lee & Shen, 2025). Embodiment-related studies were informed by anthropomorphism, social presence, uncanny or incongruent cue processing, and expectation-related accounts (Shin et al., 2019; Xia et al., 2025; Zourrig et al., 2025). Social-role and relational research drew on source credibility and parasocial perspectives (Melnychuk et al., 2024; Vo et al., 2025), social-identity and construal-level approaches (Chen et al., 2024; Mo & Wang, 2025), persuasion knowledge (van Berlo & Breves, 2025), and symbolic interactionism (Thompson et al., 2025). These frameworks do not map one-to-one onto the three dimensions; rather, they illuminate different ways in which users infer capability, presence, legitimacy, similarity, or relational meaning from digital human cues.

Across this evidence, perception–response relationships were many-to-many. Agency-related judgments were often associated with trust, credibility, usefulness, adoption, engagement, and purchase intentions. Embodiment-related judgments crossed psychological and relational domains: anthropomorphism, human-likeness, expressive behavior, social presence, and authenticity could support closeness, empathy, attachment, or positive evaluation, while incongruent or unsettling cues could contribute to eeriness, distrust, social comparison, or reduced well-being. Social-role judgments shaped what users expected from influencers, streamers, advisors, companions, and other mediated partners and were therefore connected with credibility, parasocial interaction, psychological closeness, loyalty, acceptance, and role-specific behavioral intentions. Conversely, the same response—such as trust, engagement, or purchase intention—could be associated with more than one perception dimension. The integrated pattern is therefore better characterized as a network of recurring associations than as a set of one-to-one effects.

The three response domains were similarly interrelated without forming a fixed sequence. Psychological responses captured evaluative and affective reactions, relational responses captured social connection, and behavioral responses captured intended or enacted action, but primary studies placed constructs in different analytical positions. Social presence could describe perceived presence, a relational experience, or a mediator; authenticity could be treated as an attributed quality or an evaluative response; and emotional attachment could appear as an outcome or a moderating condition. These differences are substantive rather than merely terminological because they indicate that the field has not converged on a single ordering of perception, evaluation, relationship formation, and behavior. Figure 4 therefore preserves these cross-domain relationships rather than imposing a staged process from psychological to relational to behavioral response.

The right-hand column provides an interpretive synthesis of the broader forms of engagement represented by the reported outcomes. Transactional engagement includes purchase, conversion, and related commercial responses; relational continuity includes loyalty, attachment, retention, and sustained connection; social diffusion includes recommendation, advocacy, and content sharing; and sustained adoption and participation includes continued use, acceptance, switching, and longer-term involvement. These groupings were not independently coded as a further outcome taxonomy. Their purpose is to distinguish substantively different ways in which psychological, relational, and behavioral responses can matter: purchase, attachment, recommendation, and continued use may all be favorable outcomes, but they represent different forms of involvement and may depend on different configurations of perception and response.

Finally, the feedback pathway at the bottom of Figure 4 represents an emerging pattern rather than a general feature of the evidence base. Replika users participated in training and personalizing the chatbot and invested intellectual and affective labor in constructing an imagined ideal AI partner (Pan et al., 2025); VTuber audiences participated not only as spectators but also as creators within the fandom experience (S. Lee & Lee, 2023); and avatar customization was associated with embodiment and psychological ownership (D. Y. Kim & Jang, 2025). These cases indicate that, in some contexts, user responses and participation can become inputs into subsequent representation or interaction. Feedback and co-construction are therefore presented as a tentative extension of the perception–response map, supported by a small but conceptually important subset of studies.

### 3.5. Gaps in Current Evidence Base

The evidence map revealed six interrelated gaps concerning contextual concentration, temporal depth, response scope, population diversity, theoretical integration, and design-oriented evidence.

First, the evidence base was strongly concentrated in marketing and commerce and, within those settings, in AI and virtual influencers. Thirty-four of the 51 included studies examined marketing, influencer communication, live-streaming commerce, or related consumer-facing settings, while AI and virtual influencers accounted for 28 studies. Far fewer studies addressed education and work, public or organizational communication, long-term companionship, entertainment and fandom, or non-commercial virtual-world interaction. As a result, the most developed parts of the perception–response map concern persuasive and consumer-facing digital humans, while comparable evidence remains limited for other social, institutional, and relational roles.

Second, the methodological distribution provided limited insight into change over time. Experiments and survey- or SEM-based studies accounted for most of the included research, and many examined a single encounter or cross-sectional relationship. Much of the behavioral evidence also concerned intentions rather than observed behavior. These designs are useful for identifying short-term associations and mechanisms, but they provide less evidence about how trust, attachment, role expectations, discomfort, continued use, or disengagement develop and change through repeated interaction.

Third, positive and commercially desirable responses received more attention than negative, ambivalent, or resistant outcomes. The literature included evidence on eeriness, creepiness, discomfort, reduced trust, social comparison, and uncanny effects, but these were often examined within studies primarily concerned with acceptance, engagement, or purchase. Skepticism, interaction fatigue, avoidance, role rejection, overtrust, excessive attachment, and disengagement were comparatively less visible as outcomes in their own right. This leaves the field with a stronger account of how digital humans can attract, persuade, or retain users than of when interaction becomes burdensome, inappropriate, or unwanted.

Fourth, the cultural and population scope of the evidence remained uneven. Although the included studies covered both Western and East Asian settings and included some cross-cultural evidence, many samples consisted of consumers, young adults, or users of particular social-media platforms. The extent to which authenticity, disclosure, anthropomorphism, social presence, role appropriateness, and relational expectations vary across cultures, age groups, levels of digital literacy, and prior platform experience therefore remains insufficiently established.

Fifth, the literature remained theoretically and conceptually fragmented. The included studies drew on diverse theoretical perspectives, but these were often applied within particular entity types or application contexts. Closely related constructs were also positioned differently across studies. For example, social presence could be treated as a perceived characteristic, a relational experience, or a mediator; authenticity as an attributed quality or evaluative response; and emotional attachment as an outcome or moderating condition. These differences make findings more difficult to compare across studies and point to the need for clearer specification of construct roles in future research.

Finally, the included studies provided stronger evidence about associations among digital human characteristics, perceptions, and responses than about how these insights can be translated into design and evaluation practice. Comparatively few studies tested concrete strategies intended to improve user experience, support appropriate transparency, clarify social roles, reduce discomfort, or manage relational risks across repeated interaction. Future design-oriented research could therefore move beyond testing isolated cues toward examining how combinations of agency, embodiment, and role cues can be matched to different communicative contexts and user goals.

## 4. Discussion

This scoping review synthesized 51 empirical studies on users’ perceptions of and responses to digital humans across a heterogeneous set of entity labels and application contexts. Despite this diversity, the evidence converged on a recurrent organizing pattern: users interpreted digital humans in terms of what they appeared capable of doing, how they became perceptually and socially present, and what functional or relational position they occupied. These judgments of agency, embodiment, and social role were associated with psychological, relational, and behavioral responses, but the relationships were neither one-to-one nor consistently sequential.

The principal contribution of the review is an evidence-organizing synthesis of a conceptually fragmented field rather than a new causal model. The perception–response framework separates what users attribute to digital humans from how they evaluate, relate to, and act toward them, while preserving variation in how primary studies position specific constructs. This makes it possible to compare findings across virtual influencers, streamers, avatars, advisors, companions, and other forms without assuming that the same cue or response has an identical function in every context. Three broader implications follow from this synthesis.

### 4.1. Interpretation of the Integrated Evidence

#### 4.1.1. Perception as Context-Sensitive Meaning-Making

The first implication is that users appear to make sense of digital humans through combinations of cues rather than through isolated features. Agency, embodiment, and social role answer different but connected questions: whether the entity can act meaningfully, how it is experienced as present, and what users expect from it in the interaction. The same visual or behavioral cue can therefore acquire different meanings depending on the role and setting in which it appears. Responsiveness may signal useful competence in an advisor, social attentiveness in a companion, or performance quality in a livestreaming presenter; humanlike appearance may support closeness in one context but create incongruence or creepiness in another.

This pattern is broadly consistent with the Computers Are Social Actors (CASA) perspective, which proposes that social cues from computers and interactive technologies can elicit interpersonal rules and social responses even when users know that the source is non-human (Nass & Moon, 2000; Nass et al., 1994). The reviewed evidence, however, indicates that the presence of a humanlike cue is only part of the process for digital humans. Users also interpret whether the entity appears capable, coherent, and appropriate for the role it occupies. In this sense, agency and social role help specify the context in which embodied social cues become meaningful rather than simply adding more cues to a human-likeness continuum.

The relative salience of the three dimensions also varied across application contexts. Capability and responsiveness were especially visible in recommendation, task, and advisory settings; appearance, expression, and social presence were prominent in immersive, avatar, and performance contexts; and credibility, similarity, legitimacy, and relational appropriateness became especially important in influencer, fandom, support, and companionship settings. Studies drawing on construal-level, social-identity, persuasion-knowledge, and expectation-related perspectives likewise showed that responses depended on fit between the entity, its communicative role, the product or message, and users’ expectations (Chen et al., 2024; Mo & Wang, 2025; van Berlo & Breves, 2025; Xia et al., 2025). Social role is therefore not merely another label for entity type; it provides a contextual frame through which agency and embodiment cues are interpreted.

This contextual account helps explain why greater anthropomorphism or human-likeness did not produce uniformly positive outcomes. The evidence included positive associations with closeness, engagement, and social presence, but also findings involving social comparison, eeriness, reduced authenticity, or distrust (M. Barari et al., 2025; M. Kim & Baek, 2024; Shin et al., 2019; Stein et al., 2024). Rather than supporting a simple “more humanlike is better” assumption, these findings point toward the importance of congruence among appearance, behavior, communicative style, and role expectations. Perceived digital human qualities are therefore better understood as context-sensitive interpretations of mediated cues than as direct readouts of technical features.

#### 4.1.2. Responses as Interrelated and Non-Linear

The second implication concerns the relationship among psychological, relational, and behavioral responses. These domains are analytically distinct, but the reviewed studies frequently examined them together. Trust, credibility, authenticity, enjoyment, social presence, parasocial interaction, emotional attachment, and perceived value often functioned as mechanisms linking digital human characteristics to engagement, adoption, recommendation, or purchase (H. Liu et al., 2025b; Melnychuk et al., 2024; Stein et al., 2024; Zhang et al., 2025). The evidence therefore supports a more mediated account of digital human effects than a simple feature-to-behavior model.

At the same time, the synthesis does not support a universal sequence in which psychological evaluation necessarily precedes relational connection and behavior. Constructs appeared in different positions across primary models, and favorable behavior could occur without uniformly favorable evaluation. AI influencers, for example, could generate relatively strong word-of-mouth intentions despite lower source trust (Sands et al., 2022). Conversely, attention or initial engagement does not by itself demonstrate attachment, continued use, or long-term acceptance. This distinction is particularly important because the current literature is dominated by short-term and intention-based outcomes.

The response domains are therefore best treated as different forms of consequence rather than successive stages. Psychological responses concern how users evaluate and feel about the encounter; relational responses concern whether the interaction acquires social or interpersonal meaning; and behavioral responses concern what users do or intend to do. Distinguishing these domains makes it possible to compare studies without assuming that trust, social presence, attachment, engagement, and purchase are interchangeable indicators of a single underlying state of “acceptance.” It also clarifies why the same digital human cue can produce mixed outcomes across different domains.

#### 4.1.3. Broader Forms of Engagement and Emerging Co-Construction

The third implication concerns the breadth of engagement represented in the evidence. Although marketing and commerce dominate the corpus, the reported outcomes extend beyond immediate persuasion or purchase. Transactional engagement, relational continuity, social diffusion, and sustained adoption or participation describe different ways in which digital humans become consequential to users. A purchase intention, a parasocial bond, a recommendation, and continued companion use all indicate involvement, but they differ in duration, social meaning, and the processes likely to sustain them.

This distinction matters because different forms of engagement appear to depend on different configurations of perception and response. Competence and credibility may be especially relevant to transactional decisions; similarity, social presence, and attachment may become more important to relational continuity; and recommendation or sharing may depend on credibility, entertainment value, identity expression, or the communicative value of the content. Sustained adoption is likely to require more than a favorable first impression, yet the current evidence contains relatively little longitudinal work capable of examining how these configurations change over time.

A smaller body of research further suggests that some digital human relationships are participatory rather than purely receptive. Replika users contributed intellectual and affective labor to training and personalizing the chatbot (Pan et al., 2025); VTuber audiences participated as creators within the fandom experience (S. Lee & Lee, 2023); avatar customization was associated with embodiment and psychological ownership (D. Y. Kim & Jang, 2025); and symbolic interactionist work on virtual influencers emphasized the active construction of meaning and identity around digital figures (Thompson et al., 2025). These findings are too limited to establish a general feedback mechanism, but they indicate that digital humans can function as emerging media around which users not only receive cues and form responses but also participate in shaping identities, representations, and ongoing interaction.

### 4.2. Theoretical Implications

The synthesis has three main theoretical implications. First, it provides a more differentiated account of social responding to digital humans. CASA offers an important broad explanation for why non-human technologies can evoke social judgments and interpersonal responses (Nass & Moon, 2000; Nass et al., 1994), but the present evidence shows that humanlike cues are interpreted through at least three separable questions concerning capability, embodied presence, and social position. Digital human research may therefore benefit from treating agency, embodiment, and role as complementary interpretive dimensions rather than reducing social response to anthropomorphism or human-likeness alone.

Second, the review makes visible the theoretical pluralism of the field. Mind perception and competence-related approaches illuminate whether digital humans are treated as capable or intentional actors (Toyib & Paramita, 2025; Zhang & He, 2025); anthropomorphism, social presence, uncanny-valley, and expectation-related accounts address how mediated form and cue congruence shape presence and evaluation (Shin et al., 2019; Xia et al., 2025; Zourrig et al., 2025); source-credibility and parasocial approaches explain persuasive and relational processes (Melnychuk et al., 2024; Vo et al., 2025); and social-identity, construal-level, persuasion-knowledge, and symbolic interactionist perspectives illuminate role fit, disclosure, identity, and meaning construction (Chen et al., 2024; Mo & Wang, 2025; Thompson et al., 2025; van Berlo & Breves, 2025). These theories explain different parts of the evidence rather than converging on a single overarching causal model.

The perception–response framework can therefore serve as a translation layer across these traditions. Its contribution is not to replace the theories used in primary studies, but to clarify whether a construct is functioning as something attributed to the digital human, a psychological evaluation, a relational experience, a behavioral consequence, or a mechanism linking these elements. This is particularly useful for constructs such as social presence, authenticity, and emotional attachment, which appeared in different analytical positions across studies. More explicit specification of construct roles would make theoretical claims easier to compare across entity types and contexts and would reduce the risk of treating similarly named variables as conceptually equivalent when they perform different functions.

Third, the evidence suggests a gradual expansion of digital human research from persuasion and technology acceptance toward relationality, identity, participation, and ongoing social meaning. Companions, social-support agents, VTubers, avatars, and co-created platform figures raise questions that cannot be fully captured by immediate consumer outcomes. For behavioral science and emerging-media research, this broadens the theoretical agenda from whether digital humans are persuasive or sufficiently humanlike to how users interpret them as social actors, negotiate appropriate relationships with them, and incorporate them into continuing communicative practices.

### 4.3. Limitations and Future Research

Several limitations of this review should be acknowledged. First, the review focused on English-language, peer-reviewed studies published between 2016 and 2025 and therefore excluded non-English scholarship, grey literature, industry reports, books, and other publication formats. Second, the review focused on user perceptions and responses rather than technical implementation, production processes, algorithmic design, or macro-level social impacts. Third, no formal critical appraisal was undertaken because the purpose was to map and synthesize the evidence rather than assess intervention effectiveness or estimate a pooled effect. Fourth, although two authors independently coded the extracted information and resolved disagreements through consensus, the grouping of heterogeneous constructs into broader perception dimensions and response domains necessarily involved interpretive judgment.

The composition of the evidence base also constrains the scope of the synthesis. Marketing and commerce accounted for most included studies, and AI or virtual influencers were much more frequently examined than companions, advisors, or other digital human forms. The framework is consequently most empirically developed for consumer-facing and platform-mediated contexts. Its usefulness in education, workplace communication, public communication, sustained companionship, and other non-commercial settings requires further testing rather than simple extrapolation from the current corpus.

Future research should therefore broaden both context and time horizon. Longitudinal and process-oriented studies are needed to examine how agency, embodiment, role expectations, trust, attachment, discomfort, and disengagement change through repeated interaction. Greater attention should also be given to negative and ambivalent outcomes, including skepticism, fatigue, avoidance, overtrust, excessive attachment, and relationship dissolution, rather than treating these mainly as boundary conditions around acceptance or engagement.

Greater cultural and population diversity is also needed, particularly for questions involving authenticity, disclosure, relational appropriateness, and companionship. In parallel, future studies should specify more clearly the theoretical role of central constructs and test whether those roles remain stable across entity types and media settings. Finally, theory-informed design research could move beyond isolated feature manipulations to examine how transparency, cross-modal congruence, role clarity, and interaction design jointly shape beneficial and adverse outcomes over time. Such work would help determine not only which digital human cues matter, but when, for whom, and in what communicative role they become consequential.

## 5. Conclusions

This scoping review synthesized 51 empirical studies on how users perceive and respond to digital humans across influencer, streaming, avatar, companion, and conversational agent contexts. Across a fragmented literature, three recurring perceptual questions emerged: whether the digital human appears capable of meaningful action (perceived agency), how it becomes perceptually and socially present (perceived embodiment), and what functional or relational position it occupies (perceived social role). Reported outcomes clustered into psychological, relational, and behavioral domains, but the evidence showed many-to-many and non-linear relationships rather than a single pathway from perception to response.

The main contribution of the review is an evidence-organizing framework that distinguishes what users attribute to digital humans from how they evaluate, relate to, and act toward them. This synthesis moves the field beyond treating human-likeness as the sole organizing question: digital humans are interpreted simultaneously as acting entities, embodied media representations, and participants in recognizable social roles. These interpretations were associated with different forms of trust, authenticity, social presence, parasocial connection, attachment, engagement, adoption, and commercial behavior, as well as with discomfort, distrust, social comparison, and other adverse responses. The framework should therefore be read as a map for comparing heterogeneous findings and construct roles, not as a fixed taxonomy or universal causal model.

The current evidence remains concentrated in marketing and commerce, short-term designs, and intention-based outcomes, with less attention to non-commercial settings, long-term relationships, negative or ambivalent responses, cultural variation, and design interventions. Future research can use the framework to specify more clearly which perceptions and response processes are being examined, how they are theoretically positioned, and how their relationships vary across digital human roles, user populations, and periods of interaction. For behavioral and emerging-media research, the central insight is that understanding digital humans requires more than asking how humanlike they appear; it requires examining how users interpret their capability, embodied presence, and social position, and how those interpretations become consequential in mediated communication.

## Figures and Tables

**Table 1:** Mapping user perception concepts to organizing dimensions.

Dimension	Core Question	Common Concepts	Representative Studies
Perceived agency	Can the digital human act, respond, or express intention?	Perceived intelligence; competence; autonomy; interactivity; responsiveness; communicative capability; mind perception.	(Esfahani et al., 2020; C. T. Lee & Shen, 2025; Na et al., 2025; Ranjbartabar et al., 2021; Sands et al., 2022; Toyib & Paramita, 2025; Wu & Kraemer, 2017; Zhang & He, 2025; Zourrig et al., 2025)
Perceived embodiment	How does the digital human become perceptually and socially present?	Anthropomorphism; human-likeness; social presence; authenticity; expressive embodiment; perceived creepiness.	(Chae et al., 2025; Hidayat et al., 2024; Koles et al., 2024; Liew et al., 2017; Oh et al., 2016; Rocha, 2025; Shin et al., 2019; Xu et al., 2025)
Perceived social role	What position does the digital human occupy in interaction?	Influencer identity; idol/streamer role; advisor role; companion role; perceived similarity/homophily.	(Alboqami, 2023; Hidayat et al., 2024; S. Lee & Lee, 2023; Li, 2023; Ranjbartabar et al., 2021; Rocha, 2025; Toyib & Paramita, 2024; Yoo et al., 2025)

**Table 2:** Mapping user response outcomes to response categories.

Category	Core Question	Common Outcomes	Representative Studies
Psychological responses	How do users evaluate or emotionally react to the digital human?	Trust; credibility; authenticity judgments; empathy; enjoyment/flow; perceived usefulness; eeriness; discomfort; well-being.	(Alboqami, 2023; Hidayat et al., 2024; Jiang et al., 2025; H. Kim & Park, 2024; H. Liu et al., 2025b; Muniz et al., 2024; Shin et al., 2019; Vo et al., 2025; Zhang et al., 2025)
Relational responses	Do users experience the digital human as a social or relational partner?	Parasocial interaction; social presence; psychological closeness; relational bonding; perceived similarity; homophily; emotional resonance; attachment formation; loyalty/stickiness.	(Chae et al., 2025; H. Kim & Park, 2024; Melnychuk et al., 2024; Stein et al., 2024; Vo et al., 2025; Y. Wang et al., 2025; Wu & Kraemer, 2017; Yan et al., 2024; Zhang et al., 2025)
Behavioral responses	What do users do or intend to do in relation to the digital human?	Purchase intention; following intention; subscription; engagement (likes, comments, sharing); word-of-mouth; recommendation intention; adoption; technology acceptance; switching intention; user co-creation behavior.	(Dabiran et al., 2024; Jiang et al., 2025; M. B. Khan et al., 2025; C. T. Lee & Shen, 2025; Li, 2023; Sands et al., 2022; Shao, 2025; Toyib & Paramita, 2025; Zhang et al., 2025)

## Data Availability

No new data were created or analyzed in this study. Data sharing is not applicable to this article.

## Supplementary Materials

The following supporting information can be downloaded at: https://www.mdpi.com/article/10.3390/bs16091700/s1, Table S1: Completed Data Charting Table for Included Studies; Table S2: Data Charting Form and Coding Guidance; Table S3: Full Search Strategies; File S1: Preferred Reporting Items for Systematic reviews and Meta-Analyses extension for Scoping Reviews (PRISMA-ScR) Checklist.
