# Supplementary material for: How Users Perceive and Respond to Digital Humans: A Scoping Review of Agency, Embodiment, Social Role, and Response Outcomes
Source: Behav Sci (Basel). 2026 Sep 20;16(9):1700. doi: 10.3390/bs16091700 (PMC13603254; doi:10.3390/bs16091700)
Supplement: Supplementary file 1 [file behavsci-16-01700-s001.zip › supplementary.pdf]

Supplementary Materials

How Users Perceive and Respond to Digital Humans

Table S1. Completed Data Charting Table for Included Studies

Author (Year)	Entity Label	Context	Study Design	User Perception	User Response	Key Findings	Limitations
Toyib & Paramita (2025)	AI influencer	Impact of AI influencers on consumer perception and adoption intention of electric vehicles	Two questionnaire survey (Study 1: n=129; Study 2: n=351), analyzed with Hayes' Process Macro	Perceived agency		AI influencers' competence enhances EV perceived quality and adoption intention when EVs are perceived as competent, but weakens when EVs are viewed as warm yet less competent. EV warmth promotes adoption intention through increased trust.	The focus on trust and perceived quality as mediators, without considering variables such as perceived consumer value, and the homogeneous age composition of the sample limit generalizability.
Wu & Kraemer (2017)	AI influencer	AI influencer endorsements in social media advertising, comparing AI vs. human influencers	Two-sample experiment with university students and Amazon Mechanical Turk workers, comparing avatar appearances and positive/negative language	Perceived agency	Relational responses	Positive language increases avatar preference and likeability, but neither language valence nor appearance affects perceived human control	Limited contexts and short text introductions may constrain generalizability
Li (2023)	VTuber	This study explores how viewers perceive and engage with VTubers in live-streaming contexts, focusing on how	A questionnaire survey was conducted to examine viewers' perceptions of VTuber attractiveness and their engagement behaviors during livestreaming.	Perceived social role	Behavioral responses	Parasocial interaction motivates viewers to watch VTuber livestreams. Physical, social, and task attractiveness, along with VTuber personality, livestream content, avatar design, and interaction patterns, influence viewers' engagement and social	The study used a cross-sectional questionnaire approach and focused mainly on viewer perceptions, limiting causal interpretation and broader generalization.

Author (Year)	Entity Label	Context	Study Design	User Perception	User Response	Key Findings	Limitations
		VTubers' attractiveness and parasocial interactions shape viewer choices and mediated interactions.				interactions with VTubers.	
Na et al. (2025)	Virtual streamer	Examines the mechanism through which virtual streamer characteristics influence consumers' purchase intention in live-streaming e-commerce	questionnaire survey (n=318), supplemented by fuzzy-set qualitative comparative analysis (fsQCA)	Perceived agency	Psychological responses	Virtual streamer characteristics affect purchase intention through the mediating roles of trust and affection. Perceived attractiveness is more strongly linked to affection, whereas perceived intelligence is more strongly associated with trust. In addition, two distinct configurations that lead to high purchase intention are identified.	The study focuses on a single platform and cultural context, with further research needed on consumer factors, human–virtual streamer comparisons, and cross-cultural variations.
Zourrig et al. (2025)	Humanoid virtual influencer	How humanoid virtual influencer appearance shapes social presence and consumer behavioral intention	Four online survey experiments	Perceived agency		Anthropomorphism affects social presence via animism, with warmth and competence as partial mediators, while disposition to follow recommendations links attractiveness to purchase intention.	Cross-sectional design limits causal inference; single macro Japanese influencer and Instagram-only context constrain generalizability across influencer tiers, cultures, and platforms.
Jung et al. (2022)	Conversational agent	Impact of metaphorical representations of conversational agents on worker	Between-subjects experiment (n=341) with text-based conversational agents	Perceived agency	Behavioral responses	Different human and non-human metaphors of conversational agents significantly affect worker engagement, cognitive load, intrinsic motivation, and trust.	Cross-sectional design and controlled task setting limit ecological validity; results may be affected by task specificity, cultural interpretation of

Author (Year)	Entity Label	Context	Study Design	User Perception	User Response	Key Findings	Limitations
		engagement in crowdsourcing marketplaces, based on the Great Chain of Being framework					metaphors, and gender design bias in agent representation, while the use of a generic "God" metaphor constrains clarity of competence–warmth attribution.
Sands et al. (2022)	AI social media influencer	Comparison of consumer responses to AI vs. human social media influencers, with social distance, agency, and need for uniqueness as moderating/mediating variables	Two empirical questionnaire studies	Perceived agency	Behavioral responses	The study found no significant differences between AI and human influencers in follow intention or perceived personalization. However, AI influencers elicited lower source trust but higher word-of-mouth intention. Social distance mediated these effects, while a lack of perceived agency negatively affected user responses.	Only focuses on Instagram; does not examine product category or platform differences.
Lee & Shen (2025)	AI influencer	How affordances of AI influencers shape consumer perceptions and behaviors on short-video platforms	Mixed-methods study: text mining of 12,467 user comments + questionnaire survey of 978 followers, analyzed with SEM	Perceived agency	Behavioral responses	Four types of AI affordances positively shape influencers' perceived humanness and trust, which in turn drive purchase intention and active engagement.	Cross-sectional design and Douyin-specific context limit generalizability, while broader investigation of longitudinal effects, human–AI comparisons, ethical issues, algorithmic influences, and cultural variations is needed.
Pan et al. (2025)	Social chatbot, AI companion	User immaterial labor and AI imagination in social chatbots, with the	Digital ethnography of the Replika online community	Perceived agency, Perceived social role		The study found that users, driven by the imagination of an ideal partner, invest substantial intellectual and emotional resources in training algorithms. At	Only focuses on a single product and pioneer community; generalizability is limited.

Author (Year)	Entity Label	Context	Study Design	User Perception	User Response	Key Findings	Limitations
		ventriloquism mechanism, from the perspective of immaterial labor theory				the same time, the perceived ventriloquism mechanism both facilitates and obscures the exploitation of users' intimacy and immaterial labor.	
Esfahani et al. (2020)	Anthropomorphic avatar	Effects of mindful vs. mindless anthropomorphism of platforms on consumer comprehension, attitude, and purchase intention toward really new products	Single-factor between-subjects experiment (4 levels of social cues) with 500 participants	Perceived agency		The study found that human-like avatars were preferred and elicited stronger mindless anthropomorphism, whereas interactive content did not enhance this effect. Moreover, mindless anthropomorphism improved product comprehension, attitudes toward the product, and purchase intention.	Limited utilitarian RNPs restrict generalizability across product types, domains, and hedonic contexts, with unexamined product characteristics, innovation attributes, and presentation formats potentially biasing anthropomorphism effects.
Liu et al. (2025)	AI-generated virtual streamers	Differentiated effects of virtual streamer interaction types (product-focused vs. social-focused) on purchase intention across product types	Randomized scenario experiment with two cases, total valid sample of 301	Perceived agency	Psychological responses, Relational responses, Behavioral responses	Product-focused interactions enhance purchase intention for utilitarian products via perceived value. 2. Social interactions boost purchase intention for hedonic products via social presence.	Only focuses on young consumers; limited sample representativeness; does not examine other boundary conditions.
Ranjbartabar et al. (2021)	Virtual advisor	How adaptive relational agents reduce study stress and build rapport by personalizing relational cues,	Between-subjects experiment (empathic, neutral, adaptive groups) with 111 students	Perceived agency, Perceived social role	Behavioral responses	Both the empathic and adaptive groups achieved significantly higher rapport scores than the neutral group, with the adaptive group showing the greatest increase in rapport. In addition, study stress decreased significantly among	Limited reliance on a rule-based cue elicitation approach without validation in naturalistic, longitudinal, or multimodal (e.g., non-verbal) interaction

Author (Year)	Entity Label	Context	Study Design	User Perception	User Response	Key Findings	Limitations
		addressing the cold-start problem				participants in the empathic and adaptive groups.	settings constrains ecological validity and scalability.
Liu & Zhang (2024)	Virtual streamer	Impact of virtual streamer responsiveness on consumer purchase intention in live-streaming e-commerce, with social presence as a mediator	Questionnaire survey of 432 consumers with live-stream viewing experience	Perceived agency	Psychological responses, Behavioral responses	Virtual streamer responsiveness positively affects purchase intention, with social presence playing a mediating role.	
Zhang & He (2025)	AI influencer	Impact of AI influencer endorsements on brand perception and purchase intention, from the perspective of mind perception theory	Two scenario experiments simulating real social media advertisements	Perceived agency	Psychological responses, Behavioral responses	AI influencers reduce perceived brand trustworthiness and purchase intentions through lower perceived agency	Scenario-based experimental design may limit the generalizability of the findings
Kim et al. (2025)	Virtual influencer	Differences in individual responses to social support from human vs. virtual influencers	Online experiment in the context of Instagram Stories	Perceived agency, Perceived social role	Behavioral responses	The study found no differences in evaluations of human influencers across support types. However, for virtual influencers, cognitive support resulted in higher perceived efficacy, interactivity, attractiveness, and overall attitudes. Furthermore, interactivity and social attractiveness jointly exerted a significant serial mediating effect in the virtual	Hypothetical experimental settings and Instagram-based young adult samples require further validation through real interactions, longitudinal designs, diverse populations, and cross-platform contexts.

Author (Year)	Entity Label	Context	Study Design	User Perception	User Response	Key Findings	Limitations
						influencer condition.	
Toyib & Paramita (2024)	AI fashion influencer	Effects of perceived homophily and authenticity of AI fashion influencers on recommendation adoption intention, with anthropomorphism as a mediator	Online questionnaire survey of 343 fashion consumers	Perceived agency, Perceived embodiment, Perceived social role	Behavioral responses	Homophily enhances recommendation adoption intention through the mediating effect of anthropomorphism, while authenticity moderates this indirect relationship by influencing the effect of homophily on adoption intention via anthropomorphism.	Limited examination of AI influencer stimuli and mechanisms, along with a fashion-specific context and limited sample, constrains generalizability, while ethical considerations require further exploration.
Hidayat et al. (2024)	AI influencer	Impact of AI influencer use on message reception in beauty brand campaigns on Instagram, based on audience reception theory	Quantitative questionnaire survey of 100 active Instagram users	Perceived embodiment, Perceived social role	Psychological responses	Use of AI influencers leads to declined message reception, driven by lack of trust, limited social presence, and the absence of humanistic elements. In addition, visual realism and hyperreal content create a gap between the portrayed image and reality.	Small sample size; only focuses on a single market and product category; limited generalizability.
Barari et al. (2025)	Virtual influencer	Impact of virtual influencer anthropomorphism on consumer engagement and well-being, with upward social comparison as a mediator	Mixed-methods study: content analysis of 21,745 posts from 109 virtual influencers + randomized experiment with 643 participants	Perceived embodiment	Behavioral responses	High (vs. low) anthropomorphism significantly increases user engagement but decreases well-being. These effects are mediated by upward social comparison and are further moderated by the virtual influencer’s size and perceived agency.	The study is limited by its focus on Instagram, a young consumer–based sample, and a narrow set of outcomes, which restricts generalizability across platforms, cultures, stakeholders, and product types, while unexamined contextual factors further constrain external validity.

Author (Year)	Entity Label	Context	Study Design	User Perception	User Response	Key Findings	Limitations
Xia et al. (2025)	Virtual influencer	Determinants of social media engagement with virtual influencer endorsements, based on expectation violation theory	Machine learning analysis of 9,665 virtual influencer endorsement posts on Instagram, interpreted with SHAP values	Perceived embodiment	Relational responses, Behavioral responses	Anthropomorphism is the strongest predictor of engagement and is negatively associated with it. In addition, text length, AI identity disclosure, and brand disclosure negatively affect engagement, whereas basic information cues, persona characteristics, purchase channel disclosure, and content dynamism positively enhance engagement.	Only focuses on Instagram; does not explore underlying psychological mechanisms.
Liu & Zhang (2025)	Virtual streamer	Effect of virtual streamer anthropomorphism on purchase intention in live-streaming e-commerce, with social presence as a mediator	Questionnaire survey of 362 consumers	Perceived embodiment	Psychological responses	Virtual streamer anthropomorphism positively affects purchase intention, with social presence playing a mediating role.	The study is limited by a young consumer–focused sample and a narrow product scope, which constrain generalizability, while unexamined individual differences further limit understanding of heterogeneous responses to virtual streamer interactions.
Jiang et al. (2025)	Virtual influencer	Impact of virtual influencer emotional expression on consumer behavioral intention, and its matching effect with brand stereotypes, based on ECT	Three online experiments (total n=691)		Psychological responses, Behavioral responses	Smiling expressions produce a stronger primitive emotional contagion effect, and that emotional expressions enhance brand authenticity, purchase intention, and follow intention through empathy. However, in contexts emphasizing brand competence, virtual influencers without a smile are found to be more persuasive.	Static image-based experiments and limited emotional expressions constrain ecological validity, while untested dynamic expressions and boundary conditions restrict generalizability.

Author (Year)	Entity Label	Context	Study Design	User Perception	User Response	Key Findings	Limitations
		and ELM					
Chae et al. (2025)	Virtual avatar	Effect of avatar human-likeness on psychological closeness and downstream outcomes in VR work and learning environments	Four studies + two supplementary replications, including machine learning analysis of VR recordings	Perceived embodiment	Relational responses	Higher human-likeness of avatars increases psychological closeness, which in turn leads individuals to physically move closer to the avatars. This enhanced psychological closeness further promotes trust and more favorable attitudes toward VR.	Limited focus on others' avatars and appearance-based human-likeness constrains understanding of self-avatar effects, while insufficient cross-technology validation (e.g., AR/MR) and uncanny valley analysis limit generalizability across immersive environments.
Lee et al. (2025)	AI influencer	Interaction effect of influencer type (human vs. AI) and advertising style on credibility and purchase intention, with perceived humanness as a moderator	Two experimental studies (Study 1: n=289; Study 2: n=297)	Perceived embodiment	Behavioral responses	The study found that transformational advertisements are more effective when delivered by human influencers, whereas informational advertisements perform better with AI influencers. Moreover, highly human-like AI influencers can perform as effectively as human influencers in transformational advertising contexts.	
Yan et al. (2024)	Virtual influencer	Differences in consumers' emotional attachment and benefit-seeking behavior across different types of virtual influencers	Experimental study, classifying VIs by perceived humanness and appearance realism	Perceived embodiment	Relational responses	Different types of virtual influencers generate varying levels of emotional attachment and behavioral responses, and that social presence mediates the relationship between virtual influencer type and these outcome variables.	Entertainment-focused VI posts limit generalizability across roles and industries, while unclear effects of moderately human-like VIs and boundary conditions (e.g., product type, consumer traits) restrict understanding of social

Author (Year)	Entity Label	Context	Study Design	User Perception	User Response	Key Findings	Limitations
							presence mechanisms and outcomes.
Kim & Park (2024)	Human-like virtual influencer	How human-like virtual influencers satisfy consumer experience via social presence in social media endorsements	Questionnaire survey of 364 female Instagram users		Psychological responses, Relational responses	Anthropomorphism positively influences satisfaction, while social presence does not mediate this relationship. Instead, enjoyment, perceived usefulness, flow, and credibility play significant roles in shaping the outcome.	Only focuses on female users; limited sample representativeness.
Liew et al. (2017)	Talking avatar	Effects of non-interactive talking avatars on social presence, credibility, trust, and patronage intention in e-commerce, with gender differences	Between-subjects experiment (avatar vs. no avatar control group)	Perceived embodiment		Talking avatars significantly enhance perceived social presence, but their effects on trust and patronage intention vary by gender, showing positive impacts for male participants and negative impacts for female participants. Female users tend to be more critical of avatar limitations and show a preference for textual information.	Only examines non-interactive avatars; does not compare with interactive effects.
Xu et al. (2025)	Virtual avatar	Mechanism of avatar distinctiveness on consumer purchase intention	Online experiment	Perceived embodiment	Behavioral responses	Avatar distinctiveness increases purchase intention through identification, with a serial mediation pathway from distinctiveness to identification, then to social presence, and ultimately to purchase intention. In addition, age, education level, and product type moderate these relationships.	Limited by its focus on appearance-based distinctiveness, simplified manipulation, and young samples; future research should explore broader dimensions and diverse populations.
Koles et al. (2024)	Virtual influencer	Manifestations of authenticity in the virtual	Qualitative study with interviews of 64 consumers and 11	Perceived embodiment	Psychological responses	All three types of authenticity apply to the virtual influencer context and present distinct manifestations.	Limited sample size, single cultural background, and

Author (Year)	Entity Label	Context	Study Design	User Perception	User Response	Key Findings	Limitations
		influencer context, based on the Entity-Referent Correspondence Framework	industry experts				generalizability to be verified.
Thompson et al. (2025)	Virtual influencer	How consumers construct meaning for virtual influencer identity, from the perspective of symbolic interactionism	Sequential mixed-methods study: analysis of 57,086 comments + online survey of 209 followers	Perceived embodiment, Perceived social role	Behavioral Responses	Consumers actively negotiate the reality of virtual influencers in comment sections, with a particular emphasis on anthropomorphism. Both moral and cognitive anthropomorphism enhance positive affect and engagement through the mediation of perceived shared reality.	Cross-sectional and relatively small, potentially biased sample limits causal inference and generalizability, while focusing on a single CGI human-like VI restricts cross-type applicability and theoretical scope.
Oh et al. (2016)	Virtual avatar	Effects of enhanced avatar smiling on communication experience and social presence in virtual environments	Dyadic conversation experiment with facial expression tracking, between-subjects design	Perceived embodiment		The study found that participants interacting with avatars displaying enhanced smiles use more positive words to describe their experience, as this condition induces stronger positive affect and greater perceived social presence.	Single-avatar design with only moderate realism limits generalizability, as avatar visual realism may interact with emotional expressions, potentially biasing perceived emotional intensity and interaction effects.
Rocha (2025)	Social chatbot	Multimodal semiotic signs influencing users' perception of realism in chatbots, following iconization theory	Qualitative semiotic analysis of Replika user experiences	Perceived embodiment, Perceived social role		What users perceive as "real" essentially reflects an iconization process of humanness, and that variations in voice and other multimodal semiotic features significantly shape perceptions of realism.	Only focuses on a single product; qualitative conclusions are difficult to quantify and generalize.
Mo &	Virtual	Temporal	Three experimental	Perceived		Combinations with higher temporal	Only focuses on the

Author (Year)	Entity Label	Context	Study Design	User Perception	User Response	Key Findings	Limitations
Wang (2025)	influencer	alignment effect between influencer type and brand heritage narration on luxury consumption, based on construal level theory	studies with middle-income Chinese consumers	social role		alignment (virtual influencer with present narration and human influencer with past narration) generate more favorable consumer responses, with an incremental mindset mediating this positive effect. In addition, construal level moderates the effect on incremental mindset.	Chinese market and luxury category; limited generalizability.
Wang et al. (2025)	Virtual influencer	Effects of virtual influencer characteristics on purchase intention among Chinese Gen Z consumers, with brand equity and emotional attachment as moderators	Questionnaire survey of 403 Gen Z consumers, analyzed with PLS-SEM	Perceived social role	Psychological responses, Relational responses	All five core characteristics significantly and positively influence purchase intention, while brand equity moderates the effects of controllability and congruence. In addition, emotional attachment amplifies the effects of interactivity, parasocial relationship, and source credibility, although some moderating effects are not statistically significant.	Only focuses on the Gen Z group; cross-sectional data cannot verify dynamic causality.
Lee & Lee (2023)	VTubers, virtual avatar	Effects of perceived VTuber role and platform type on audience presence, immersion, and fan creation behavior	Mixed-methods study: survey of 1,795 audience members + interviews with 10 voluntary creators	Perceived social role		Both platform type and perceived role affect presence and immersion, while the motivational context for creation also considerably influences content output.	Only focuses on a single VTuber case; limited generalizability.
Shao (2025)	Virtual streamer	Determinants of consumers' switching	Online questionnaire survey, analyzed with NCA, ANN, and fsQCA	Perceived social role	Behavioral responses	Six distinct configurations of factors leading to switching intention are identified, each with unique causal	Only focuses on the UK market; cultural differences require further

Author (Year)	Entity Label	Context	Study Design	User Perception	User Response	Key Findings	Limitations
		intention from human to virtual streamers, integrating innovation barriers, shopping motivations, and personalities				paths.	verification.
Chen et al. (2024)	Virtual live streamer	Influence mechanism of anthropomorphism on consumers' willingness to accept virtual live streamers, based on social identity theory and construal level theory	×2 between-subjects scenario experiment (n=214)	Perceived social role	Behavioral responses	Anthropomorphism positively influences acceptance willingness, with psychological distance and trust sequentially mediating this relationship. In addition, product type moderates the effect, such that the serial mediation is significant for utilitarian products but not for hedonic products.	Only uses scenario experiments and effects in real scenarios remain to be verified.
Dabiran et al. (2024)	AI companion	Investigated how virtual influencers influence followers' perceptions, parasocial relationships, and purchase intention in marketing.	Conducted a questionnaire-based survey with 430 respondents to examine the effects	Perceived embodiment	Relational responses, Behavioral responses	Parasocial relationships increased purchase intention, and influencer–product congruence strengthening the credibility–purchase intention relationship.	Only focused on the virtual influencer marketing context, relying on cross-sectional self-reported survey data, which may limit causal inference and the generalizability of findings across different cultural settings and application contexts.
Alboqami (2023)	AI influencer	Causal recipes for customer	fsQCA analysis of 683 consumers	Perceived social role	Psychological responses,	No single factor is sufficient to generate high trust, and instead	Only focuses on the Saudi retail context; strong

Author (Year)	Entity Label	Context	Study Design	User Perception	User Response	Key Findings	Limitations
		trust in AI influencers in retail, based on complexity theory			Relational responses	identified five distinct configurations of factors that can lead to high trust.	cultural and industry limitations.
Yoo et al. (2025)	Virtual influencer	Effects of textual social cues on virtual influencer credibility and purchase intention, with anthropomorphism as a moderator	Randomized between-subjects experiment (n=265), analyzed with PLS-SEM	Perceived social role	Relational responses	Textual social cues directly influence attractiveness and homophily, which subsequently predict credibility and purchase intention, and that incorporating such cues helps overcome the novelty effect while fostering long-term follower relationships.	Only examines textual cues without multimodal factors; fictional influencers have limited ecological validity.
Kim & Baek (2024)	Virtual influencer	Effects of virtual influencer attributes on perceived creepiness, authenticity, emotional attachment, and word-of-mouth intention	Questionnaire survey of US social media users	Perceived embodiment, Perceived social role	Psychological responses	Attitude homophily positively influences perceived creepiness, while most similarity dimensions enhance perceived authenticity. Both perceived creepiness and authenticity, in turn, significantly affect emotional attachment and word-of-mouth intention.	Only focuses on US users; cross-cultural generalizability to be verified.
Chesney et al. (2016)	Virtual avatar	Effects of user personality and social value orientation on avatar-mediated friendship in virtual worlds	Field study with 179 participants, combined with server log data	Perceived social role		Personality and values influence both the number of friends and active friendship behavior, while only conscientiousness exhibits a homophily effect. However, neither personality nor values affect the number of incoming friend requests.	Only examines a single virtual world; does not explore in-depth psychological mechanisms.
van Berlo & Breves	Virtual influencer	Effects of prominence of	Two between-subjects experiments (Study 1:	Perceived social role	Psychological responses	Prominent disclosure reduces perceived credibility and leads to	Only focuses on female users; only examines the

Author (Year)	Entity Label	Context	Study Design	User Perception	User Response	Key Findings	Limitations
(2025)		virtual influencer nature disclosure on consumer perceptions and brand responses, based on the persuasion knowledge model	n=245; Study 2: n=429)			more negative brand responses, with source credibility mediating the relationship between disclosure prominence and brand outcomes. However, product digitality does not moderate these effects.	immediate effect of single disclosure.
Stein et al. (2024)	Virtual influencer	Differences in parasocial interaction with real vs. virtual influencers, with similarity and humanness as mediators	Pre-registered experiment with natural stimuli (n=179)	Perceived embodiment	Relational responses	The study found no significant difference in overall parasocial interaction between the two types, while two opposing mediating effects were observed: virtual influencers exert a stronger direct effect, but their lower perceived humanness and similarity suppress this advantage.	Only a single-exposure experiment; does not examine long-term interaction effects.
Melnychuk et al. (2024)	Virtual influencer	Mechanism of follower stickiness to virtual influencers, based on parasocial interaction theory and source credibility theory	Online questionnaire survey of 302 Instagram followers		Psychological responses, Relational responses	Source credibility positively influences parasocial interaction, which in turn significantly enhances engagement and user stickiness.	Only focuses on Instagram; cross-sectional data cannot verify causal direction.
Khan et al. (2025)	Virtual avatar	Factors influencing	Questionnaire survey of 770 participants, analyzed	Perceived embodiment	Psychological responses,	Perceived similarity, social presence, and anthropomorphism	Rapid technological changes necessitate

Author (Year)	Entity Label	Context	Study Design	User Perception	User Response	Key Findings	Limitations
		users' intention to adopt avatars as digital self-representations on social networking platforms	with SmartPLS		Behavioral responses	all positively affect trust and enjoyment, which in turn shape adoption intention.	further investigation of avatar-mediated interactions across evolving technological contexts.
Kim & Jang (2025)	Virtual avatar	Effect of avatar customization on purchase intention for virtual fashion items, with serial mediation of embodiment and psychological ownership	Two experiments simulating metaverse avatar customization	Perceived embodiment	Behavioral responses	The findings show a serial effect of customization on purchase intention via embodiment and psychological ownership, and this effect is amplified by higher avatar human-likeness.	Only focuses on fashion categories; experimental scenarios differ from real metaverse environments.
Shin et al. (2019)	Virtual avatar	Uncanny valley effect of avatar realism and animacy on trustworthiness and friendship decisions in virtual social networks	×2 between-subjects online experiment (n=134)	Perceived embodiment	Psychological responses	Hyper-realistic and animated avatars evoke stronger feelings of eeriness, which in turn reduce perceived trustworthiness, and this diminished trustworthiness ultimately shapes friendship acceptance decisions.	Only examines simple avatar forms; effects in complex interaction scenarios remain to be verified.
Liu et al. (2025)	Virtual idol	Emotional dynamics of fan engagement in virtual idol endorsements, case study of Luo Tianyi	Phenomenological approach: in-depth interviews + netnography of online communities		Psychological responses, Relational responses, Behavioral responses	The study found that authenticity, entertainment value, and aesthetic appeal are key antecedents of emotional attachment, while intimate interactions amplify emotional resonance and further promote brand recognition and purchase behavior. In addition, fans	Single case study; qualitative conclusions are difficult to quantify and generalize.

Author (Year)	Entity Label	Context	Study Design	User Perception	User Response	Key Findings	Limitations
						actively participate in co-constructing the virtual influencer ecosystem.	
Rungruangjit et al. (2024)	Human-like virtual influencer	Impact of human-like virtual influencer-generated content types on consumers' willingness to follow and purchase intention	Quantitative study of 650 consumers, analyzed with PLS-SEM		Behavioral responses	Emotional appeal content has the strongest effect on both outcome variables, while disclosure of sponsored content does not have a significant effect on follow willingness.	Only focuses on the Thai market and Instagram; cross-cultural generalizability to be verified.
Vo et al. (2025)	Virtual influencer	How virtual influencers drive customer engagement and brand loyalty, based on parasocial theory and source credibility model	Questionnaire survey (n=406) + semi-structured interviews, analyzed with PLS-SEM		Psychological responses, Relational responses	Trustworthiness and authenticity positively influence engagement, while parasocial interaction serves as a major driver of affective reactions and engagement. In contrast, virtual influencer attractiveness is not directly associated with engagement.	Limited VI attributes and minority sampling constrain generalizability, while unexplored factors restrict explanatory completeness and external validity.
Muniz et al. (2024)	Virtual influencer	Effect of disclosing virtual influencers' non-human nature on brand trust, with cross-cultural differences	Three within-subjects experimental studies (US: n=75, n=101; Brazil: n=83)	Perceived embodiment	Psychological responses	Disclosure reduces anthropomorphism, credibility, and brand trust; however, the indirect effect on brand trust is not supported among Brazilian consumers, suggesting the presence of cultural differences.	Only examines immediate effects of single disclosure; cultural dimensions are not comprehensively explored.

Table S2. Data Charting Form and Coding Guidance

Note. Table S2 describes the data charting form and coding guidance used to extract, classify, and organize information from the included studies. The resulting coded data are presented in Table S1.

Item	What to record	Coding guidance
Author (Year)	Record author names and publication year.	Use the citation format that will appear in the manuscript. If there are two studies by the same author in the same year, label them consistently as 2023a and 2023b after checking the reference list.
Entity Label	Record the exact term or terms used by the authors to describe the entity.	Do not normalize the authors' terminology in this column. This column is for mapping terminology. If the authors use several terms, record all major terms.
Context	Record a concise description of the setting, platform, application domain, or empirical situation in which users encounter or respond to the digital human.	Use the information reported in the study and retain relevant contextual detail. List the primary context first when more than one setting is involved.
Study Design	Record the study design or research method.	Use the method stated by the authors. If the paper uses more than one method, list all major methods. If it is not empirical, code as "Conceptual article" or "Review."
User Perception Dimensions	Record the user perception concepts examined or discussed in the study.	Record the study-specific perception concepts during extraction. In Table S1, these concepts are organized under one or more of the three dimensions listed below. If no dimension is assigned in the final charting table, use an em dash.
User Response Outcomes	Record the psychological, relational, attitudinal, or behavioral outcomes examined or discussed.	Record the outcomes examined or discussed in the source study. In Table S1, these outcomes are organized under one or more of the three response categories listed below. If no category is assigned in the final charting table, use an em dash.
Key Findings	Summarize the main findings or arguments relevant to this review.	Use one to three sentences. Focus only on findings related to digital humans, user perceptions, and user responses. Do not summarize the entire paper if parts are irrelevant to the review. If the study is conceptual, summarize the main argument.
Limitations	Record limitations, gaps, or unresolved issues that matter for this scoping review.	Do not simply copy all limitations from the article. Record only those that help identify gaps relevant to this review. Include both limitations stated by the authors and limitations identified by the review team. Make clear if the gap is reviewer identified by writing "Reviewer noted."

Perception organizing dimensions

Organizing dimension	Core perceptual question	Common concepts in reviewed studies	Boundary rule
Perceived agency	Can the digital human act, respond, or express intention?	perceived autonomy, responsiveness, interactivity, intentionality, intelligence, AI control, human control, …	Code here when the concept concerns action capacity, responsiveness, autonomy, or attribution of control
Perceived embodiment	How does the digital human become perceptually and socially present?	anthropomorphism, human likeness, realism, facial expression, voice, multimodality, social presence, …	Code here when the concept concerns form, sensory presentation, humanlike appearance, modality, or embodied presence
Perceived social role	What position does the digital human occupy in interaction?	influencer, companion, performer, brand representative, public persona, …	Code here when the concept concerns the role, relational position, or communicative function attributed to the digital human

Response categories used in Table S1

Response category	Description
Psychological responses	Psychological, evaluative, or emotional responses reported in the included study.
Relational responses	Responses concerning social or relational connection with the digital human.
Behavioral responses	Actions or behavioral intentions reported in relation to the digital human.

Note. These descriptions clarify the three response labels used in the final charting table; they do not add a separate coding layer or additional study findings.

Table S3. Full Search Strategies

Scopus – Perception

TITLE-ABS-KEY("digital human*" OR "virtual human*" OR metahuman* OR "virtual influencer*" OR "CGI influencer*" OR "AI influencer*" OR "synthetic infl uencer*" OR VTuber* OR "virtual idol*" OR "virtual character*" OR "virtual agent*" OR "embodied conversational agent*" OR "conversational agent*" OR " AI companion*" OR "digital avatar*" OR "virtual avatar*" OR avatar* OR"AI digital human*" OR "AI anchor*" OR "virtual anchor*" OR "virtual KOL*" OR "virt ual actor*" OR "virtual host*" OR "virtual spokesperson*" OR "virtual brand officer*" OR "virtual customer service*" OR "virtual tour guide*" OR "virtual narr ator*" OR "virtual streamer*" OR "synthetic persona*" OR "CGI character*" OR "animated agent*" OR "relational agent*" OR "social agent*")
AND
TITLE-ABS-KEY("user perception" OR anthropomorphism OR humanness OR "human likeness" OR "social presence" OR "perceived agency" OR trust OR credibility OR authenticity OR interactivity)

Scopus – Response

TITLE-ABS-KEY("digital human*" OR "virtual human*" OR metahuman* OR "virtual influencer*" OR "CGI influencer*" OR "AI influencer*" OR "synthetic infl uencer*" OR VTuber* OR "virtual idol*" OR "virtual character*" OR "virtual agent*" OR "embodied conversational agent*" OR "conversational agent*" OR " AI companion*" OR "digital avatar*" OR "virtual avatar*" OR avatar* OR"AI digital human*" OR "AI anchor*" OR "virtual anchor*" OR "virtual KOL*" OR "virt ual actor*" OR "virtual host*" OR "virtual spokesperson*" OR "virtual brand officer*" OR "virtual customer service*" OR "virtual tour guide*" OR "virtual narr ator*" OR "virtual streamer*" OR "synthetic persona*" OR "CGI character*" OR "animated agent*" OR "relational agent*" OR "social agent*")
AND
TITLE-ABS-KEY("user response" OR "user acceptance" OR satisfaction OR "user engagement" OR "human-machine interaction" OR "HCI" OR participatio n OR "behavioral intention" OR "purchase intention" OR "adoption intention")

Web of Science – Perception

TS=("digital human*" OR "virtual human*" OR metahuman* OR "virtual influencer*" OR "CGI influencer*" OR "AI influencer*" OR "synthetic influencer*" O R VTuber* OR "virtual idol*" OR "virtual character*" OR "virtual agent*" OR "embodied conversational agent*" OR "conversational agent*" OR "AI compani on*" OR "digital avatar*" OR "virtual avatar*" OR avatar* OR"AI digital human*" OR "AI anchor*" OR "virtual anchor*" OR "virtual KOL*" OR "virtual actor*"

OR "virtual host*" OR "virtual spokesperson*" OR "virtual brand officer*" OR "virtual customer service*" OR "virtual tour guide*" OR "virtual narrator*" OR " virtual streamer*" OR "synthetic persona*" OR "CGI character*" OR "animated agent*" OR "relational agent*" OR "social agent*")
AND
TS=("user perception" OR anthropomorphism OR humanness OR "human likeness" OR "social presence" OR "perceived agency" OR trust OR credibility OR authenticity OR interactivity)

Web of Science – Response

TS=("digital human*" OR "virtual human*" OR metahuman* OR "virtual influencer*" OR "CGI influencer*" OR "AI influencer*" OR "synthetic influencer*" O R VTuber* OR "virtual idol*" OR "virtual character*" OR "virtual agent*" OR "embodied conversational agent*" OR "conversational agent*" OR "AI compani on*" OR "digital avatar*" OR "virtual avatar*" OR avatar* OR"AI digital human*" OR "AI anchor*" OR "virtual anchor*" OR "virtual KOL*" OR "virtual actor*" OR "virtual host*" OR "virtual spokesperson*" OR "virtual brand officer*" OR "virtual customer service*" OR "virtual tour guide*" OR "virtual narrator*" OR " virtual streamer*" OR "synthetic persona*" OR "CGI character*" OR "animated agent*" OR "relational agent*" OR "social agent*")
AND
TS=("user response" OR "user acceptance" OR satisfaction OR "user engagement" OR "human-machine interaction" OR "HCI" OR participation OR "beha vioral intention" OR "purchase intention" OR "adoption intention")

EBSCO – Perception

((TI ("digital human*" OR "virtual human*" OR metahuman* OR "virtual influencer*" OR "CGI influencer*" OR "AI influencer*" OR "synthetic influencer*" O R VTuber* OR "virtual idol*" OR "virtual character*" OR "virtual agent*" OR "embodied conversational agent*" OR "conversational agent*" OR "AI compani on*" OR "digital avatar*" OR "virtual avatar*" OR avatar* OR"AI digital human*" OR "AI anchor*" OR "virtual anchor*" OR "virtual KOL*" OR "virtual actor*" OR "virtual host*" OR "virtual spokesperson*" OR "virtual brand officer*" OR "virtual customer service*" OR "virtual tour guide*" OR "virtual narrator*" OR " virtual streamer*" OR "synthetic persona*" OR "CGI character*" OR "animated agent*" OR "relational agent*" OR "social agent*") OR AB("digital human*" OR "virtual human*" OR metahuman* OR "virtual influencer*" OR "CGI influencer*" OR "AI influencer*" OR "synthetic influencer*" OR VTuber* OR "virtual i dol*" OR "virtual character*" OR "virtual agent*" OR "embodied conversational agent*" OR "conversational agent*" OR "AI companion*" OR "digital avatar* " OR "virtual avatar*" OR avatar* OR"AI digital human*" OR "AI anchor*" OR "virtual anchor*" OR "virtual KOL*" OR "virtual actor*" OR "virtual host*" OR "vi rtual spokesperson*" OR "virtual brand officer*" OR "virtual customer service*" OR "virtual tour guide*" OR "virtual narrator*" OR "virtual streamer*" OR "s ynthetic persona*" OR "CGI character*" OR "animated agent*" OR "relational agent*" OR "social agent*"))

AND
(TI ("user perception" OR anthropomorphism OR humanness OR "human likeness" OR "social presence" OR "perceived agency" OR trust OR credibility OR authenticity OR interactivity) OR AB("user perception" OR anthropomorphism OR humanness OR "human likeness" OR "social presence" OR "perceived agency" OR trust OR credibility OR authenticity OR interactivity)))

EBSCO – Response

((TI ("digital human*" OR "virtual human*" OR metahuman* OR "virtual influencer*" OR "CGI influencer*" OR "AI influencer*" OR "synthetic influencer*" OR VTuber* OR "virtual idol*" OR "virtual character*" OR "virtual agent*" OR "embodied conversational agent*" OR "conversational agent*" OR "AI companion*" OR "digital avatar*" OR "virtual avatar*" OR avatar* OR"AI digital human*" OR "AI anchor*" OR "virtual anchor*" OR "virtual KOL*" OR "virtual actor*" OR "virtual host*" OR "virtual spokesperson*" OR "virtual brand officer*" OR "virtual customer service*" OR "virtual tour guide*" OR "virtual narrator*" OR " virtual streamer*" OR "synthetic persona*" OR "CGI character*" OR "animated agent*" OR "relational agent*" OR "social agent*") OR AB("digital human*" OR "virtual human*" OR metahuman* OR "virtual influencer*" OR "CGI influencer*" OR "AI influencer*" OR "synthetic influencer*" OR VTuber* OR "virtual idol*" OR "virtual character*" OR "virtual agent*" OR "embodied conversational agent*" OR "conversational agent*" OR "AI companion*" OR "digital avatar* " OR "virtual avatar*" OR avatar* OR"AI digital human*" OR "AI anchor*" OR "virtual anchor*" OR "virtual KOL*" OR "virtual actor*" OR "virtual host*" OR "virtual spokesperson*" OR "virtual brand officer*" OR "virtual customer service*" OR "virtual tour guide*" OR "virtual narrator*" OR "virtual streamer*" OR "synthetic persona*" OR "CGI character*" OR "animated agent*" OR "relational agent*" OR "social agent*"))
AND
(TI ("user response" OR "user acceptance" OR satisfaction OR "user engagement" OR "human-machine interaction" OR "HCI" OR participation OR "behavioral intention" OR "purchase intention" OR "adoption intention") OR AB ("user response" OR "user acceptance" OR satisfaction OR "user engagement" OR "human-machine interaction" OR "HCI" OR participation OR "behavioral intention" OR "purchase intention" OR "adoption intention")))
